# Beyond geometric constraint: dynamic fluid-lattice-activated topochemical polymerization

**DOI:** 10.1093/nsr/nwag360

**Published:** 2026-06-12

**Authors:** Bin Mu, Xiao Luo, Juanjuan Wei, Linqi Yang, Huanjun Lu, Wei Tian

**Affiliations:** Shaanxi Key Laboratory of Macromolecular Science and Technology, Xi’an Key Laboratory of Hybrid Luminescent Materials and Photonic Device, MOE Key Laboratory of Material Physics and Chemistry under Extraordinary Conditions, School of Chemistry and Chemical Engineering, Northwestern Polytechnical University, Xi’an 710072, China; Shaanxi Key Laboratory of Macromolecular Science and Technology, Xi’an Key Laboratory of Hybrid Luminescent Materials and Photonic Device, MOE Key Laboratory of Material Physics and Chemistry under Extraordinary Conditions, School of Chemistry and Chemical Engineering, Northwestern Polytechnical University, Xi’an 710072, China; Shaanxi Key Laboratory of Macromolecular Science and Technology, Xi’an Key Laboratory of Hybrid Luminescent Materials and Photonic Device, MOE Key Laboratory of Material Physics and Chemistry under Extraordinary Conditions, School of Chemistry and Chemical Engineering, Northwestern Polytechnical University, Xi’an 710072, China; Shaanxi Key Laboratory of Macromolecular Science and Technology, Xi’an Key Laboratory of Hybrid Luminescent Materials and Photonic Device, MOE Key Laboratory of Material Physics and Chemistry under Extraordinary Conditions, School of Chemistry and Chemical Engineering, Northwestern Polytechnical University, Xi’an 710072, China; Jiangsu Key Laboratory of Micro and Nano Heat Fluid Flow Technology and Energy Application, School of Physical Science and Technology, Suzhou University of Science and Technology, Suzhou 215009, China; Shaanxi Key Laboratory of Macromolecular Science and Technology, Xi’an Key Laboratory of Hybrid Luminescent Materials and Photonic Device, MOE Key Laboratory of Material Physics and Chemistry under Extraordinary Conditions, School of Chemistry and Chemical Engineering, Northwestern Polytechnical University, Xi’an 710072, China

**Keywords:** self-assembly, liquid crystals, polymerization, photochemistry, cycloaddition

## Abstract

Topochemical polymerization affords lattice-directed polymers with high structural fidelity, but its scope is limited by the need for precise, static preorganization of reactive groups—an intrinsically fragile outcome of subtle intermolecular packing. Here we introduce dynamical fluid lattices into topochemical design to circumvent these geometric constraints, achieving near-quantitative monomer conversion even without ideal preorganization. Employing core-shell columnar liquid crystals, rapid molecular fluctuations within the fluid lattices enable temporary proximity of reactive sites that activate topochemical reactions. Meanwhile, the anisotropic columnar architecture directs chain growth into well-defined linear helical polymers. Despite their covalent backbones, the resulting polymers undergo temperature-dependent, dissipative depolymerization that enables complete monomer regeneration. We integrate fluid-lattice-activated topochemical polymerization into skin-attachable films, demonstrating temporary information encryption via fluorescence imaging at body temperature. This strategy expands the design space of topochemical synthesis and suggests opportunities in regenerable polymers and flexible wearable technologies.

## Teaser

Liquid crystals harness dynamic fluid lattice motion to activate topochemical polymerization beyond conventional geometric constraints, yielding recyclable polymers with dissipative fluorescence readout.

## INTRODUCTION

**Figure 1.**
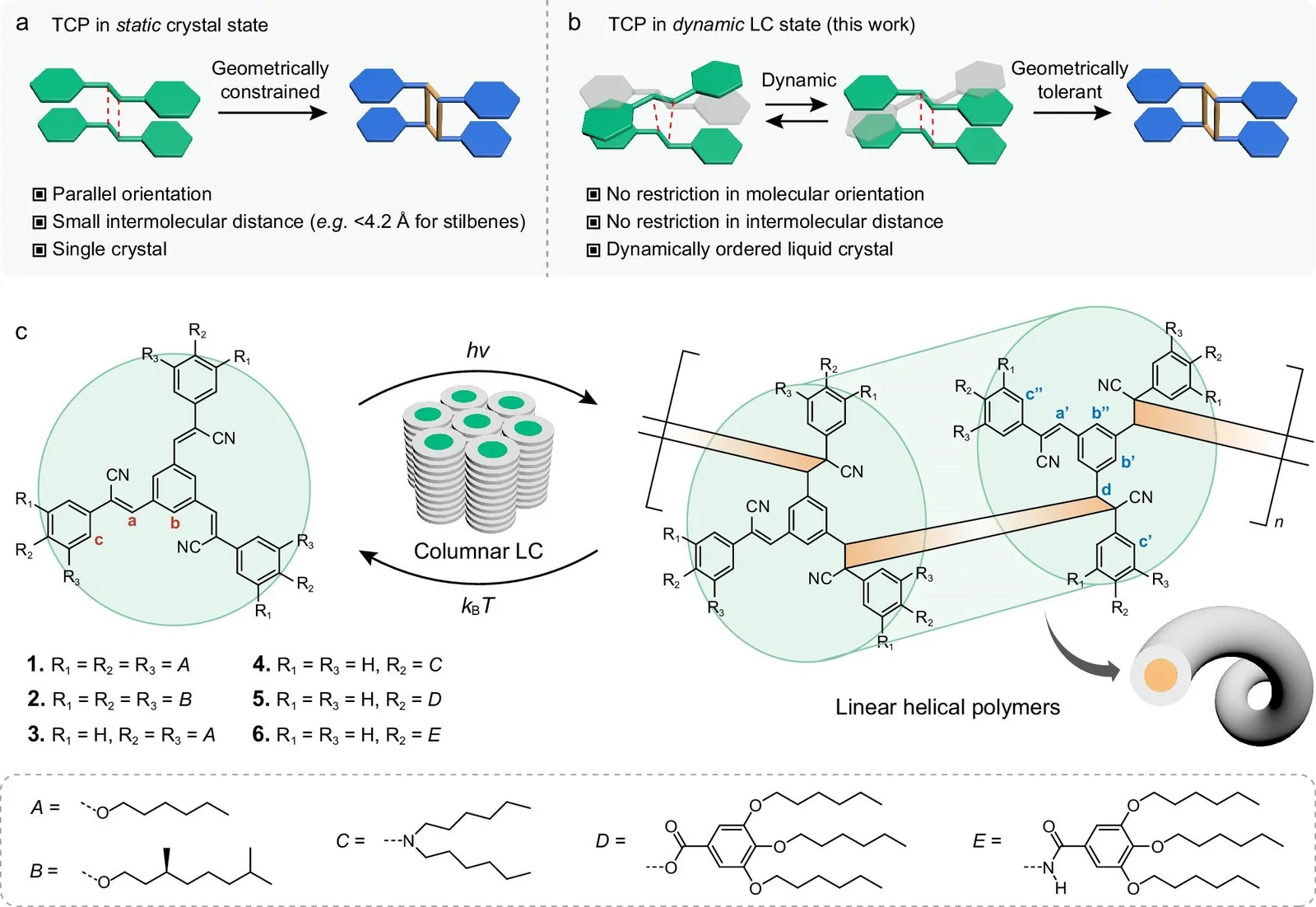
General considerations of topochemical polymerization (TCP). (a) Schematic representation of the stringent constraints required for topochemical reaction in rigid crystal lattices. (b) Flexible conditions for topochemical reaction in LC state due to the dynamic molecular motions. (c) Chemical structures of the three-armed CS discotic LC monomers **1–6**. The self-assembled core-shell columnar LC structures promote axial TCP via [2 + 2] cycloaddition of the CS cores, while the aliphatic shells prevent intercolumnar crosslinking, leading to the formation of linear helical polymers. The letters (a, a’, b, etc.) indicate protons with ^1^H NMR chemical shifts marked in Fig. 2c.

Solvent-free methodologies for materials synthesis and processing represent critical aspects in the pursuit of environmentally sustainable chemistry [1–5]. Topochemical polymerization (TCP), dictated by the geometric arrangement of reactant molecules within crystal lattices, has emerged as a powerful strategy for solid-state polymer synthesis [6–13]. This approach enables the controlled formation of stereoregular polymers that are often difficult to achieve through traditional solution-phase chemistry, where high molecular freedom permits diverse reaction pathways [14–24]. Recent decades have witnessed burgeoning advancements in the design of organic crystals tailored specifically for TCP, facilitating their utility in sustainable materials, responsive systems and energy-related applications [25–34]. Typically, TCP relies on the preorganization of molecules within crystal lattice, wherein reactive groups are placed in close proximity and oriented to facilitate optimal orbital overlap (Fig. 1a) [35–39]. Specific functional groups or intermolecular interactions have been used to promote favorable crystalline arrangements [40–46]. However, the exact packing of molecules in crystals is challenging to manipulate in a predictable fashion due to the intricate interplay of weak intermolecular interactions [47,48]. As such, the design of suitable molecular crystals for TCP is largely empirical, as even a slight chemical modification can unpredictably influence the carefully arranged molecular packing [49–51]. Therefore, the lack of broad applicability presents a substantial obstacle for the practical utilization of TCP.

To circumvent these limitations and advance the development of a more versatile TCP framework, it is necessary to devise a method that accommodates a broader range of permissible molecular alignments in ordered molecular solids. A promising strategy is to endow the rigid lattices with controlled fluidity, wherein dynamic molecular motions can facilitate self-adjustment of reactive geometries, thereby promoting geometrically tolerant TCP. In this regard, liquid crystals (LCs), which combine the anisotropic order of crystals with the dynamic mobility of liquids, have emerged as promising candidates for developing evolutive fluid lattices [52–57]. Such a dynamically ordered molecular system is compatible with rapid molecular fluctuations such as rotation and translation while maintaining structural integrity. Consequently, reactive molecules in LC state may temporarily allow them to approach the requisite proximity to activate topochemical reactions (Fig. 1b). Meanwhile, the anisotropic organization of LC lattices can guide the reaction pathways, enabling regio- and stereo-selective transformations comparable to those observed in crystals. Thus, the dynamic control of molecular arrangements within LC-based fluid lattices offers an effective means to circumvent the stringent geometric constraints typically imposed on TCP. We have recently synthesized various columnar LCs [58,59] and achieved precise control over supramolecular polymerization process [60] and assembly architectures [61,62]. However, despite the promising potential of TCP in LCs, this effectuation has yet to be demonstrated experimentally.

To address this gap, here we report a strategy that harnesses a dynamic ordering of molecules within fluid lattices of LC phases to activate TCP beyond conventional geometric constraints, enabling high polymerization efficiency and broad monomer compatibility. Specifically, we designed a series of three-armed cyano-stilbene (CS) discotic monomers (**1**–**6**) that self-assemble into core-shell columnar LC structures. In these fluid lattices, π-stacked aromatic CS cores provide a dynamic microenvironment for directing TCP, while aliphatic shells prevent undesired intercolumnar (interchain) crosslinking (Fig. 1c). Despite the geometrical constraints of helical stacking within the columnar phases, light-activated [2 + 2] cycloaddition proceeds with near-quantitative efficiency due to the molecular adaptability inherent in the dynamic LC lattices. The one-dimensional (1D) columnar architecture promotes directional chain growth along the columnar axis, resulting in efficient TCP into linear helical polymers. These polymers exhibit dissipative behavior, undergoing spontaneous and complete depolymerization via cycloreversion to regenerate the monomers. Leveraging this dissipative property, we demonstrate that fluid-lattice-activated TCP provides an efficient approach to temporary information encryption in skin-attachable films through fluorescence visualization at body temperature, thereby offering new avenues for applications in flexible optoelectronics.

## RESULTS AND DISCUSSION

Achieving TCP in LC state requires the incorporation of reactive mesogenic units capable of maintaining the molecular organization required for activating solid-state reactivity. In this context, stilbene derivatives are particularly attractive candidates, having been widely utilized in topochemical reactions through light-induced intermolecular [2 + 2] cycloaddition [35–39]. However, such reactions are limited by Schmidt’s principle [63], which requires that reactive olefinic units must be arranged in parallel with an intermolecular distance of less than 4.2 Å (Fig. 1a), thereby limiting their broader applicability. Moreover, the design of stilbene monomers suitable for TCP should meet two key conditions: (1) incorporation of multiple olefinic sites to enable polymer chain growth, and (2) the ability to form 1D architectures to direct linear polymerization. To address these requirements, we designed a series of three-armed CS monomers (**1**–**6**) with varying peripheral substituents (Fig. 1c and Figs S1–S3). Obviously, their discotic geometries facilitate regular stacking into ordered 1D columns, which further organize into hexagonal columnar LC lattices. Specifically, monomer **1** undergoes a sequential phase transition from a rectangular columnar crystalline (Cr) phase to an ordered hexagonal columnar LC (Col_ho_), followed by a disordered hexagonal columnar LC (Col_hd_), and finally to an isotropic (Iso) liquid phase (Figs S4–S10). At low temperatures, the discotic geometry of **1** promotes crystallization into the Cr phase. Upon heating above 9°C, the Cr melts into Col_ho_, where molecules adopt a helical packing arrangement within the ordered columnar structure. Further heating above 120°C disrupts intracolumnar order, resulting in Col_hd_ phase with reduced molecular correlation. At temperatures exceeding 191°C, the system transitions to Iso phase. The observed sequence of columnar phases and associated stacking behavior closely resembles those of conventional discotic LCs incorporating polycyclic aromatic mesogens [64,65]. The presence of multiple thermotropic columnar phases not only demonstrates the feasibility of conducting TCP in the LC state but also enables control over the TCP process through modulation of columnar structural parameters.

**Figure 2.**
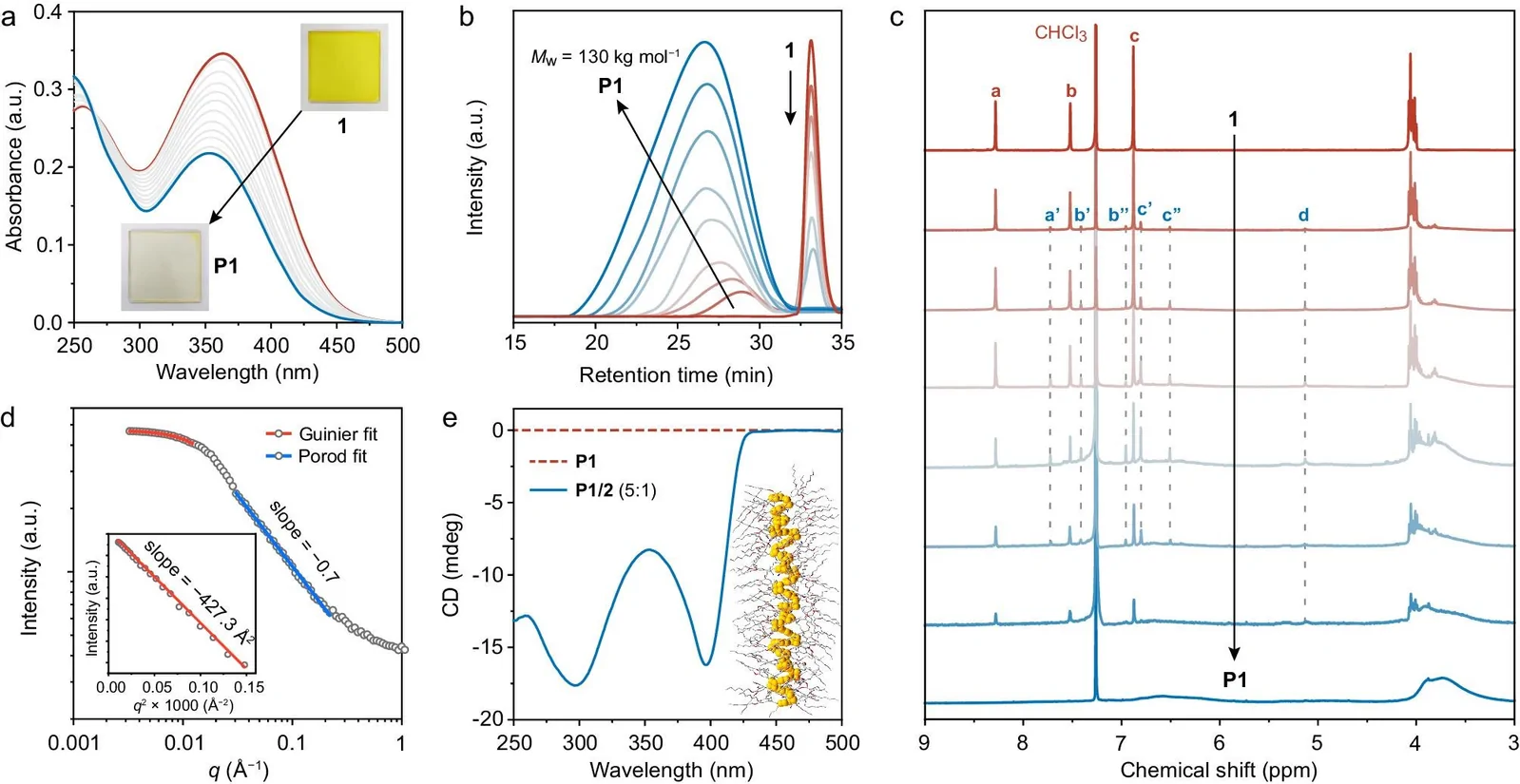
Light-activated TCP of **1** at 75°C in Col_ho_ mesophase. (a) UV-vis absorption spectra recorded during TCP of **1** in thin film upon 365 nm light irradiation for 60 min, accompanied by the corresponding real images (inset). (b) Evolution of GPC traces for TCP of **1** upon extending irradiation duration, demonstrating increased molecular weight alongside with the increased conversion. (c) ^1^H NMR spectra in CDCl_3_ (∼8 mg ml^−1^) illustrating the chemical structure changes of **1** upon light-activated TCP in LC film state over 14 h at 2-h intervals, with proton assignments shown in Fig. 1c. (d) SANS profile of **P1** in CDCl_3_, associated with Guinier and Porod fit, illustrating the extended rod-like long chain conformation. (e) CD spectra of **P1** and **P1/2** (**1/2** = 5:1 molar ratio) in dichloromethane solution (∼0.1 mg ml^−1^). Inset shows the energy-optimized structure of **P1** (16-mer) calculated at the GFN2-xTB level. The main-chain backbones are shown with space filling models for illustrating their helical conformation.

TCP was first conducted for **1** at 75°C in Col_ho_ phase. A thermally pressed LC thin film (∼5 μm thick) was irradiated with 365 nm ultraviolet (UV) light at the maximum absorption band, during which the initial yellow coloration of the film gradually faded over 24 h. Ultraviolet-visible (UV-vis) absorption spectra of the thin film before and after polymerization, as well as the images of the yellow-colored monomer film and nearly colorless polymer film, are shown in Fig. 2a. Upon irradiation, the UV-vis spectra exhibited a decrease in absorption at 360 nm (hypochromic effect), accompanied by a blue shift, reflecting the disruption of conjugated CS chromophores during TCP. The presence of an isosbestic point at 263 nm suggests an equilibrium between the polymeric **P1** and monomeric **1** state, which shifts entirely to the polymeric state with extended irradiation. Structural evolution during polymerization was analyzed by small-angle X-ray scattering (SAXS) and polarized optical microscopy (POM), revealing a loss of long-range order and a contraction in columnar dimensions, attributable to the disruption of rigid CS cores that hinder tight molecular packing (Fig. S11). Unlike conventional TCP in single crystals, which typically yields poorly soluble polymers, the LC-mediated TCP produces polymers with good solution processability. As shown in Fig. 2b, gel permeation chromatography (GPC) shows a decrease in the monomer peak and a corresponding increase in a broadened peak at shorter retention times upon irradiation. This evolution is characteristic of a step-growth polymerization behavior, reflecting the formation of higher molecular weight polymer species as the conversion progresses (Fig. S12 and Table S1). The resulting high-molecular-weight polymer reached a weight-average molecular weight (*M*_w_) of 130 kg mol^−1^ with nearly quantitative monomer conversion (>99%). In comparison, most of the light-induced TCP remains incomplete as the newly formed polymer at the crystal surface obstructs further light penetration into the bulk. Here, the TCP achieved in LC thin films enables quantitative efficiency, making the approach promising for practical and scalable applications.

Then, the chemical structure change of **1** during light-activated TCP was systematically studied. By dissolving the corresponding films in CDCl_3_ after increasing irradiation durations, the ^1^H NMR spectra exhibited obvious upfield shifts in the aromatic and methylenic ether proton signals (Fig. 2c), indicating the formation of high-molecular-weight polymer species [34,42,66,67]. At the early stage of polymerization, the spectra remained well-resolved, with the characteristic peaks of **1** gradually diminishing, while a new set of peaks emerged and intensified. Notably, peak **d** is characteristic of cyclobutane moieties formed via intermolecular [2 + 2] cycloaddition. These newly appearing signals were assigned to the cyclobutane-linked polymer structure, with chemical shifts closely matching those previously reported for head-to-head CS dimers [68,69]. Consistent with the GPC results, NMR analysis also revealed an almost quantitative monomer conversion (>99%), and polymer growth proceeds through a bidirectional step-growth process rather than random branching or crosslinking. The chain conformation of **P1** was examined by small-angle neutron scattering (SANS) in a CDCl_3_ solution (Fig. 2d) [70,71]. Fitting the curve at the low-*q* region (*qR*_g_ < 1) using the Guinier model yielded an average radius of gyration (*R*_g_) of 35.8 Å (inset in Fig. 2d). Analysis of the curve in the Porod regime (*qR*_g_ > 1) provided a slope of 0.7, which is consistent with the fractal exponent of an extended rod-like conformation for the polymer chain. Molecular simulations for **P1** further supported this finding, revealing a helical, straight long-chain backbone surrounded by bulky alkyl side chains (inset in Fig. 2e). The final polymer is expected to be racemic, as no chiral bias is present, consistent with the flat circular dichroism (CD) spectrum (Fig. 2e). The helical nature of the polymer was experimentally validated by copolymerizing **1** with **2** bearing chiral alkyl chains (Figs S13 and S14). The chirality of **2** was transferred and amplified within the coassembled Col_ho_ structure, resulting in a preferential left-handed helical columnar arrangement. Upon TCP in Col_ho_ phase, the helical handedness was retained even in solution (Fig. 2e), accounting for the observed extended helical conformation of the cyclobutane-linked backbone. Therefore, the LC assembly of **1** enables light-activated [2 + 2] cycloaddition between adjacent CS molecules, resulting in a cyclobutane-linked polymeric product consisting entirely of helical, extended long chains.

To assess the broad applicability of TCP activated by dynamic molecular ordering, we extended the monomer structures from **1**, with nine alkoxy chains, to include derivatives bearing six alkoxy chains (**3**), three dialkylamino chains (**4**), and three wedge-shaped trialkyloxyphenyl groups linked via ester bond (**5**) (Fig. 1c). All these monomers exhibited thermotropic phase transitions from columnar Cr or glassy (G) phases to Col_ho_ before entering the Iso liquid (Fig. S1). Despite structural variations, monomers **3–5** underwent efficient TCP upon irradiation in LC state, analogous to **1**, yielding high-molecular-weight linear polymers (Figs S15–S17). These results demonstrate the broad applicability of dynamic molecular ordering in facilitating TCP beyond strict geometric prerequisites. In contrast, control experiments using monomer **1** in its low-temperature Cr phase (∼0°C), as well as single crystals of the same three-armed CS mesogenic core, where molecular dynamics are constrained by rigid lattice packing, showed no reactivity even after 24 h of irradiation (Fig. S18). These observations highlight the essential role of dynamic molecular motions in activating TCP and overcoming the geometric constraints that hinder polymerization in more rigid or crystalline environments.

**Figure 3.**
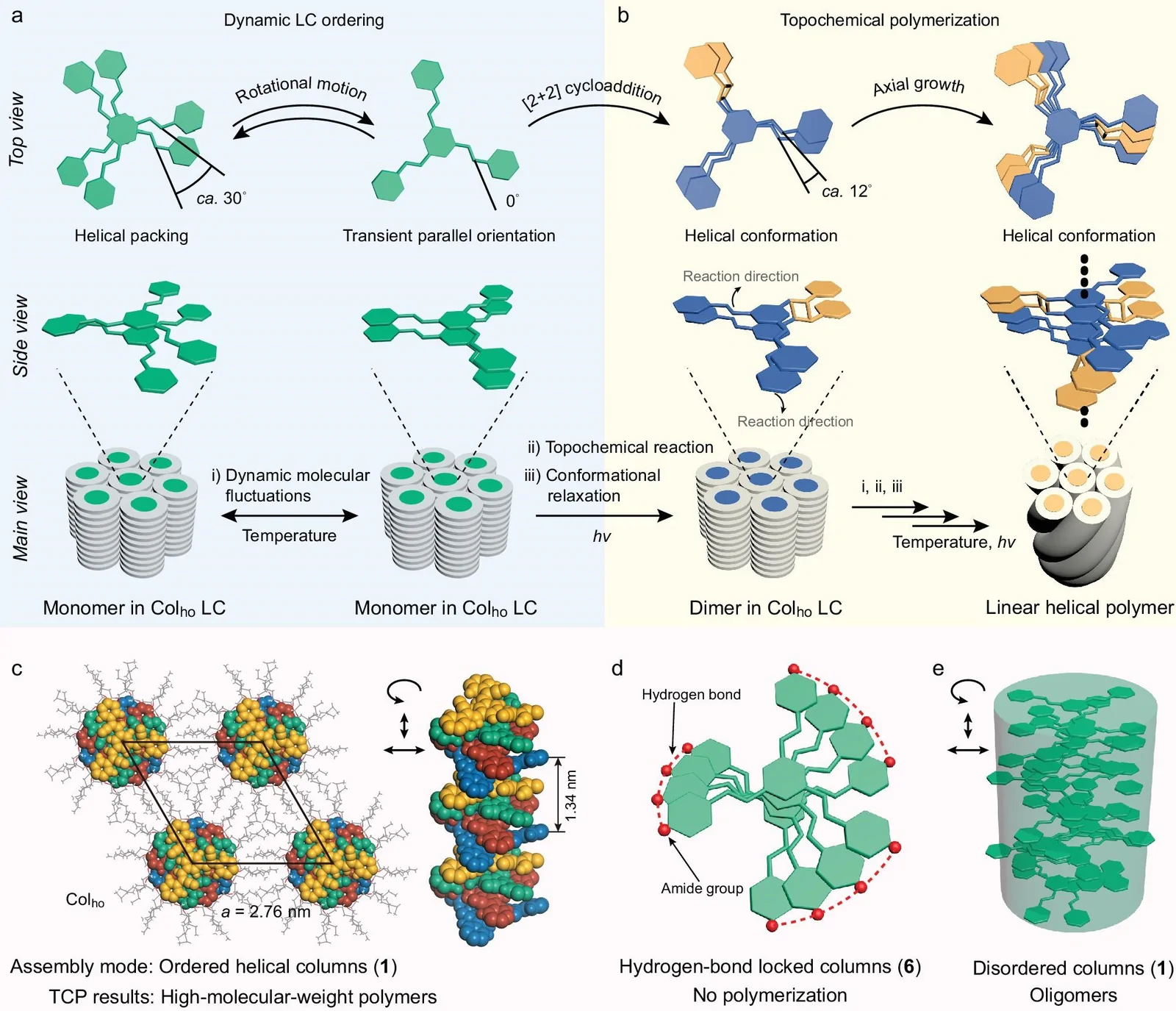
Mechanistic insight into the TCP in LC state. (a) Monomer **1** forms Col_ho_ phase through intracolumnar helical packing with a twist angle of ∼30°, and its dynamic molecular fluctuations, such as rotational motion, enable transient parallel alignment of adjacent molecules. (b) Intermolecular [2 + 2] cycloaddition of one of the arms followed by conformational relaxation to minimize energy, and bidirectional chain growth along the columnar axis to form linear helical polymers. (c) Packing model of the Col_ho_ structure of **1**, with π-conjugated aromatic cores and alkyl chains shown with space-filling and stick models, respectively. The black rhombus represents the unit cell. (d) Schematic illustration of the columns locked through intermolecular hydrogen-bonding interactions in the Col_ho_ phase of **6**. (e) Proposed intracolumnar disordered packing in the Col_hd_ phase of **1**.

The mechanism of above-mentioned TCP can be rationalized through the dynamic fluid behavior of molecules within the columnar LC lattices (Fig. 3a and b). Monomer **1** adopts a Col_ho_ mesophase characterized by helically stacked columns, where neighboring molecules within a column are laterally rotated by ∼30°, leading to a helical stacking with four molecules per pitch (Fig. 3c). According to Schmidt’s principle [63], intermolecular [2 + 2] cycloaddition is typically prohibited due to unfavorable orbital alignment. However, the dynamic molecular fluctuations in the LC state, such as rotational motion, can induce temporal eclipsed stacking of adjacent molecules (Fig. 3a). When the distance between two cofacial reactive units falls below 4.2 Å, intermolecular cycloaddition occurs via one of the three CS arms. The resulting dimer undergoes rapid conformational relaxation to relieve the ring strain associated with the substituted cyclobutane moieties (Fig. 3b). Density functional theory calculations indicate that the relaxed dimer adopts a reduced twist angle of ∼12°, while the remaining CS arms become misaligned, with inter-arm distances exceeding 4.2 Å (Fig. S19), thereby precluding further cycloaddition within the same dimer due to steric hindrance. Nonetheless, these unreacted CS arms remain available for interaction with neighboring molecules along the same column. As the reactive CS units are aligned at both ends of the growing assembly, each newly formed cyclobutane linkage preserves the directional alignment of adjacent reactive sites, enabling further cycloaddition to proceed from both termini. Consequently, the observed growth arises from a bidirectional step-growth polymerization along the columnar axis (Fig. 3b). Notably, only two of the three CS arms participate in the cycloaddition due to intrachain steric hindrance and interchain segregation of alkyl shells. This selective reactivity directs directional axial growth, facilitating the formation of high-molecular-weight linear polymers.

To further elucidate the role of dynamic molecular mobility in TCP, we performed light-activated TCP of CS derivatives with varying degrees of dynamic freedom. Monomer **6** (Fig. 1c), bearing three wedge-shaped trialkyloxyphenyl groups linked via amide bond, also formed a Col_ho_ phase. However, intermolecular hydrogen-bonding between the amide moieties significantly restricted the dynamic motion of CS cores within the LC state (Fig. 3d and Fig. S20). As a result, TCP was effectively suppressed, and no polymerization was observed under irradiation, reaffirming the critical importance of molecular dynamics for activating TCP. We next performed light-activated TCP of **1** in Col_hd_ phase (Fig. 3e). Although both Col_ho_ and Col_hd_ phases share similar hexagonal columnar lattices, the intermolecular correlations within the columns significantly reduces by the Col_ho_-Col_hd_ transition (Figs S4–S10 and S21). By contrast to the helically ordered Col_ho_ phase, the increased conformational flexibility in Col_hd_ phase permits not only intermolecular cycloaddition but also intramolecular *Z-E* isomerization of the CS arms (Fig. S22). The resulting highly twisted conformers hinder efficient molecular packing, thereby limiting the extent of cycloaddition and restricting polymer chain propagation. As a result, TCP in Col_hd_ phase predominantly yields low-molecular-weight oligomers with *M*_w_ below 10 kg mol^−1^ (Fig. S23), whereas TCP in Col_ho_ phase consistently affords high-molecular-weight polymers with little dependence on temperature (Fig. S24). Overall, dynamic molecular motions can relieve geometric constraints to activate topochemical reactivity; however, excessive molecular freedom, akin to that in solution-phase reactions, may promote undesired side processes [68,72]. As a consequence, a high degree of molecular order with adequate intermolecular correlations while maintaining moderate molecular dynamics is more suitable for facilitating efficient TCP and achieving high-molecular-weight polymers.

**Figure 4.**
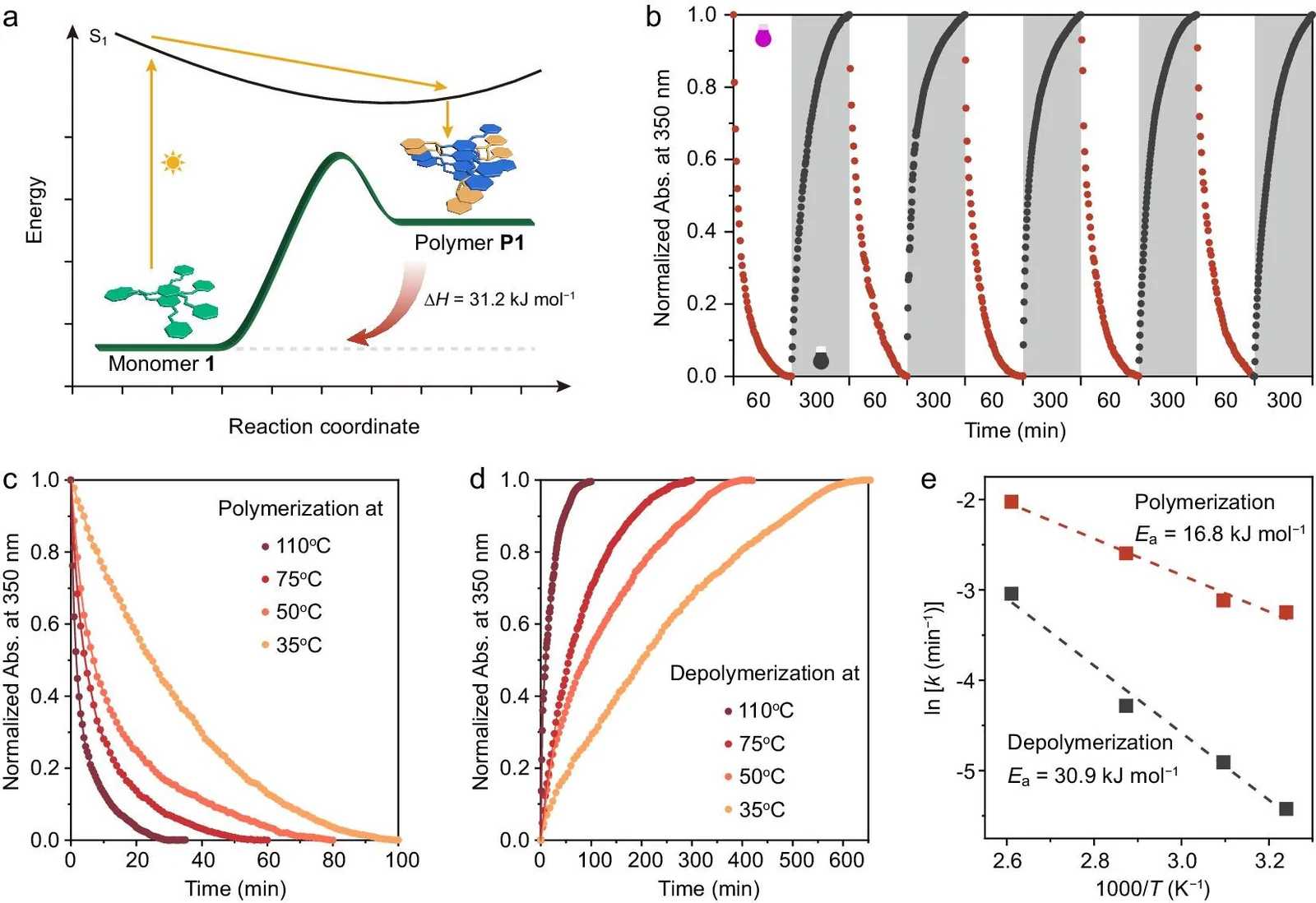
Dissipative properties of TCP controlled by temperature. (a) An energy diagram illustrating the energy levels during TCP. The ∆*H* associated with the triggered cycloreversion is estimated through DSC analysis. (b) Normalized absorbance (Abs.) at 350 nm for five cycles of dissipative TCP processes between the monomeric **1** and polymeric **P1** over time. The white regions represent light-induced polymerization, while the gray regions indicate thermal depolymerization. (c) Temperature-dependent polymerization kinetics of **1** under UV irradiation, as evidenced by the decrease in absorbance at 350 nm. (d) Temperature-dependent depolymerization kinetics of **P1**, indicated by the increase in absorbance at 350 nm. (e) Plots of the reaction rate constant (*k*) versus temperature, with dashed lines representing fits to the Arrhenius model.

Building on the implementation of TCP in fluid lattices, we next examined the properties of the resulting polymers formed in such a dynamically ordered molecular system. The cyclobutane-linked polymers are metastable and susceptible to undergo cycloreversion to monomers due to the significant ring strain of substituted cyclobutane moieties. Given that all monomers share the same mesogenic core and undergo similar TCP to form cyclobutene-linked polymers, we expect that comparable reversibility is achievable in principle, although the regeneration efficiency may vary depending on substituent effects. As a representative proof-of-concept, we focused primarily on monomer **1** due to its wide LC temperature range. This reversion occurs through thermal relaxation, releasing the energy difference between thermodynamically stable **1** and metastable **P1** (Fig. 4a). Differential scanning calorimetry (DSC) analysis indicates an enthalpy change (∆*H*) of approximately 31.2 kJ mol^−1^ (Fig. S25). Restoration of the original Col_ho_ structure was verified by POM and SAXS (Fig. S26), and the molecular identity of the monomer recovered by depolymerization of **P1** was confirmed by ^1^H NMR analysis (Fig. S27), demonstrating near-quantitative regeneration of **1**. The reversible and dissipative process of **1** was then tracked by UV-vis absorption spectroscopy, demonstrating good reversibility as evidenced by the variations in normalized absorbance at 350 nm over five polymerization-depolymerization cycles (Fig. 4b and Fig. S28). Notably, the lifetime of the dissipative TCP was highly sensitive to temperature during both polymerization and depolymerization. At elevated temperatures, the time required for light-induced TCP process was obviously reduced (Fig. 4c), with the half-life decreasing from 35 min at 35°C to 5 min at 110°C. Similarly, the half-life for the thermal depolymerization in the dark decreased from 200 min at 35°C to 10 min at 110°C (Fig. 4d). Kinetic modeling using the first-order rate law, ln[1/(1−*α*)] = *kt* (where *α* is the conversion and *k* is the rate constant), yielded nearly linear fits for both processes (Figs S29 and S30). The temperature dependence of *k* followed the Arrhenius equation, ln *k* = ln *k*_0_ − *E*_a_/*RT*, affording activation energy (*E*_a_) of 16.8 kJ mol^−1^ for polymerization and 30.9 kJ mol^−1^ for depolymerization (Fig. 4e). These kinetic results confirm the dissipative nature of the TCP system. The relatively large ∆*H* value compared to the difference in *E*_a_ is likely attributable to the contribution of liquid crystallization enthalpy (Fig. S25). Collectively, these results suggest that the forward polymerization is possibly governed by a kinetic preference in which cycloaddition proceeds faster than the corresponding retrocycloaddition, while the resulting molecular weights are, to some extent, influenced by the thermodynamic equilibrium between monomeric and polymeric states. Overall, we have established a light-fueled dissipative TCP system, involving light-induced TCP from **1** to **P1** and thermal depolymerization back to **1**, with programmable lifetimes. While dissipative behavior has been extensively studied in supramolecular and polymeric systems [73–78], this work provides an example of light-fueled dissipative polymerization cycle in ordered LC state, offering an avenue for the design of non-equilibrium LC materials.

**Figure 5.**
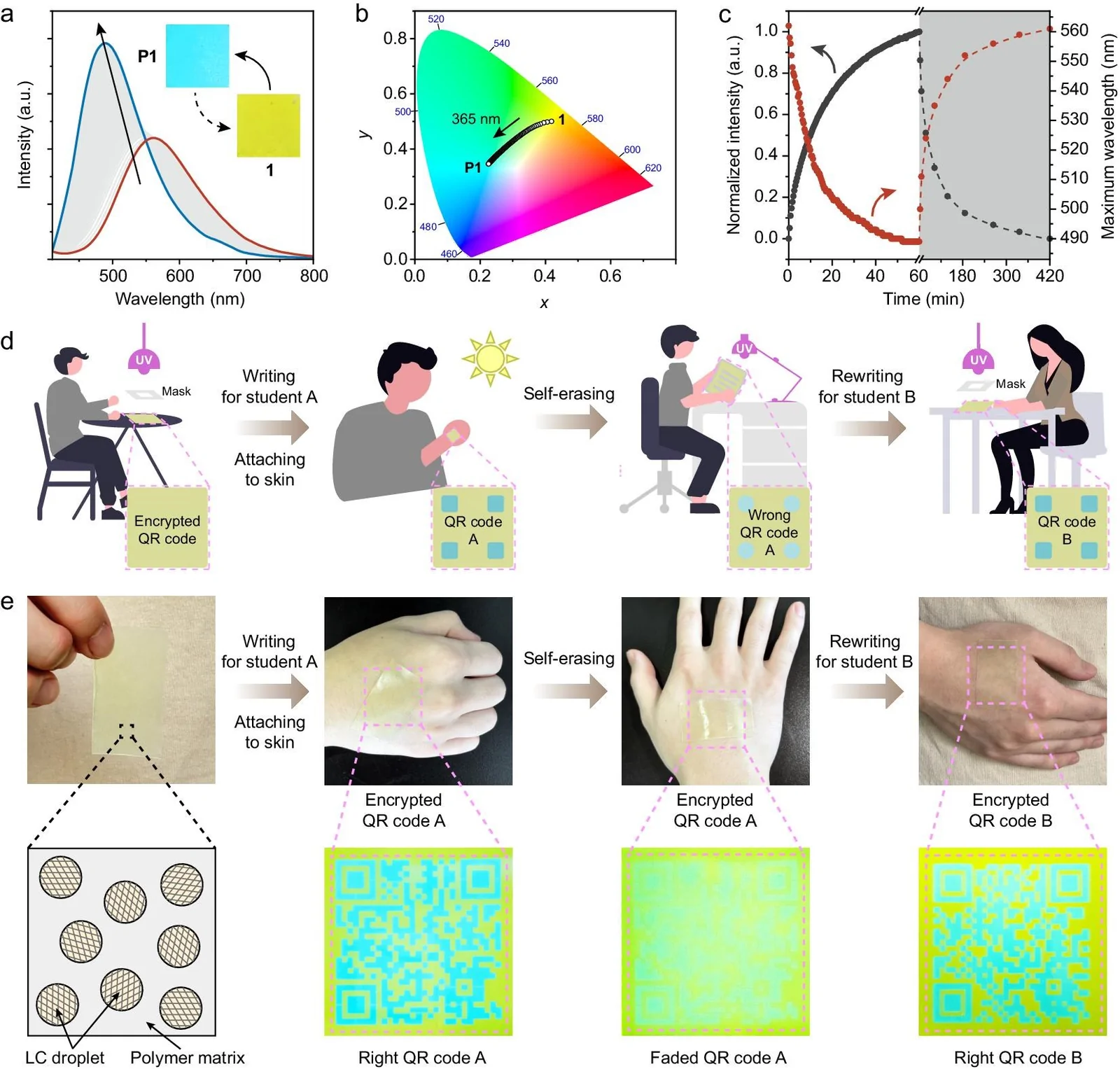
Skin-attachable fluorescence visualization via dissipative TCP. (a) Fluorescence emission spectra evolution of **1** under 365 nm light irradiation. (b) CIE 1931 chromaticity diagram illustrates the color switching trace under irradiation. (c) Normalized emission intensity at 500 nm and emission maximum during the dissipative cycle between monomeric **1** and polymeric **P1**. The white parts represent the light-induced polymerization process, and the gray parts represent the depolymerization process. (d) Schematic illustration of the practical application of dissipative TCP for information encryption through body temperature. (e) Images of the actual use of TCP in a polymer-dispersed LC film. A QR code encoding basic information for student A was written by TCP using a photomask, and the information can be easily encrypted by attaching it to human skin. Time-dependent erasure of the information was achieved via thermal depolymerization at body temperature. After complete erasure, a new QR code encoding student B’s information was rewritten.

Dissipative systems that change properties upon exposure to stimuli offer the interesting prospect of dynamically adaptive functions. In particular, coupling dissipative TCP with fluorescence processes could be a promising path toward new emergent behaviors [78–80]. The rationally designed CS compounds exhibit excellent solid-state fluorescence, in particular, monomer **1** displays an absolute quantum yield of 0.51, offering opportunities to make the dissipative process visualizable. Upon polymerization of **1**, the fluorescence emission undergoes a pronounced hypsochromic shift, with the emission maximum progressing from 562 to 488 nm and the overall intensity increasing (Fig. 5a). This spectral evolution is attributed to the disruption of aromatic π-stacking interactions, which reduces aggregation and enhances radiative decay. Correspondingly, the emission color transitions from yellow to cyan, as evidenced by the CIE 1931 chromaticity diagram (Fig. 5b) and visually supported by photographic images (Fig. S31). Notably, the emission spectra substantially recover to its original state in the absence of light (Fig. S32), indicating a reversible photochromic fluorescence switching process. A comparative analysis of emission intensity and wavelength (Fig. 5c) demonstrates that the fluorescence changes align with the absorbance variations observed in the dissipative system. Hence, the fluorescence color change serves as a more distinct way to track the dissipative dynamics of TCP. Importantly, this time-dependent fluorescence variation could be exploited for advanced applications such as information encryption, enabling programmable emission color tuning possibilities.

Leveraging the fluorescence-visualized dissipative TCP, we developed a proof-of-concept demonstration of temporary information encryption in real life triggered by body temperature. Figure 5d presents a schematic illustration of employing dissipative TCP as an information security technology. A QR code containing student-specific information was written on a film via photopatterned TCP, allowing the information to be scanned under specific conditions. When the film is adhered to human skin, thermal depolymerization upon heating at body temperature leads to gradual erasure of the encoded information. This erasure process effectively renders the information inaccessible and allows the film to be reused by different individuals. Figure 5e demonstrates a real-world implementation of this concept using a flexible film fabricated through polymer-dispersed LC technology (Fig. S33), where reversible polymerization-depolymerization cycles occur within LC droplets. The QR code containing student A’s information was written on the film by selective TCP using a photomask, producing spatially defined regions that were invisible under ambient light. Upon UV illumination, the irradiated (polymerized) areas exhibited cyan fluorescence, while unreacted (monomer) regions retained a yellow emission, enabling the QR code to be clearly visualized and successfully scanned. When the film was attached to student A’s hand, body heat gradually triggered depolymerization, causing a progressive fading of the emission pattern. The information remained accessible for up to four hours due to sufficient fluorescence contrast (Fig. S34). Prolonged attachment duration resulted in substantial depolymerization, effectively erasing the emission pattern and rendering the information unreadable. Notably, the erasing process relies only on mild human-body heat, and that prolonged contact should be performed under non-occlusive conditions and discontinued immediately if any discomfort occurs. After complete erasure of the pattern over 10 h, new information for student B was recorded on the same film using a different photomask. When this new pattern was exposed to student B’s body heat, a similar time-dependent erasure process occurred, again driven by dissipative TCP (Fig. S34). This cycle illustrates the feasibility of a reversible, body temperature-responsive encryption system based on dissipative TCP, enabling secure, temporary information display and multi-user reusability.

## CONCLUSION

We present an efficient strategy that exploits dynamic molecular motion in fluid lattices to overcome the geometrical constraints that typically limit TCP in rigid crystalline environments. Employing CS-derived discotic monomers that self-assemble into core-shell helical columnar LCs, we achieve near-quantitative [2 + 2] photocycloaddition within the confined aromatic cores. The reactivity is triggered even in the absence of ideal preorganization: temporary molecular proximity within the fluid LC lattice is sufficient to activate topochemical cycloaddition. The 1D columnar architecture facilitates directional chain growth along the columnar axis, yielding linear helical polymers with well-defined architectures and high structural fidelity. Importantly, the TCP process is dissipative, with the formed polymers undergoing complete depolymerization via cycloreversion, thereby enabling chemical recyclability of the monomers. This reversible polymerization-depolymerization cycle is accompanied by distinct fluorescence color changes, providing a built-in optical readout for real-time monitoring and adding a layer of functional complexity. By integrating this dissipative TCP system into LC films, we further demonstrate proof-of-concept applicability in human-interactive temporary information encryption. Although this study focuses on a specific class of columnar LCs, the underlying design principle—dynamical fluid-lattice-activated TCP—is potentially generalizable to other types of mesophases, offering avenues for further control over polymer dimensionality, sequence, and beyond. Thus, our findings establish a versatile framework for the synthesis of structurally ordered yet functionally tunable polymer materials, advancing the scope of solid-state synthetic methodologies.

## METHODS

Additional experimental details including materials and measurements, full synthetic details accompanied with molecular characterization data, structural characterization, absorption and emission spectra, and theoretical simulations are available in the supplementary data.

Light-Activated TCP Methods. Monomers (∼10 mg) were thermally pressed onto a quartz substrate (5 cm × 5 cm) at a temperature above its clearing point, then cooled to specified temperature (e.g. 75°C) to form a thin film. The film was annealed at this temperature for 10 min and then placed under a 365 nm UV lamp until the film became colorless and nearly transparent, yielding the polymer product. The resulting polymer was either peeled from the substrate as a solid or dissolved in an appropriate solvent (e.g. CDCl_3_, tetrahydrofuran) prior to characterization.

## Supplementary Material

nwag360_Supplemental_File
