# Supplementary material for: Beyond geometric constraint: dynamic fluid-lattice-activated topochemical polymerization
Source: Natl Sci Rev. 2026 Jun 12;13(16):nwag360. doi: 10.1093/nsr/nwag360 (PMC13499222; doi:10.1093/nsr/nwag360)
Supplement: Supplementary file 1 [file nwag360_supplemental_file.pdf]

Supplementary data

Beyond geometric constraint: dynamic fluid-lattice-activated topochemical polymerization

Bin Mu[1], Xiao Luo[1], Juanjuan Wei[1], Linqi Yang[1], Huanjun Lu[2] and Wei Tian[1,*]

[1]Shaanxi Key Laboratory of Macromolecular Science and Technology, Xi'an Key Laboratory of Hybrid Luminescent Materials and Photonic Device, MOE Key Laboratory of Material Physics and Chemistry under Extraordinary Conditions, School of Chemistry and Chemical Engineering, Northwestern Polytechnical University, Xi'an, 710072, China.

[2]Jiangsu Key Laboratory of Micro and Nano Heat Fluid Flow Technology and Energy Application, School of Physical Science and Technology, Suzhou University of Science and Technology, Suzhou 215009, China.

*Corresponding author. E-mail: happytw_3000@nwpu.edu.cn

Supplementary Methods

Materials. Methyl gallate (>98%, TCI), 1-bromohexane (>98%, TCI), lithium aluminum hydride (1 mol/L in tetrahydrofuran, Aladdin), trimethylsilyl cyanide (97%, Innochem), and benzene-1,3,5-tricarbaldehyde (>98%, TCI) were used as received. All other chemical reagents were commercially available and used as received.

Measurements. ^{1}H NMR and ^{13}C NMR spectra in solution were obtained from Bruker Avance 400 instrument. Multiplicities are denoted as follows: s = singlet, d = doublet, t = triplet, and m = multiplet. Mass spectra were recorded on a Bruker Autoflex II matrix-assisted laser desorption/ionization time-of-flight (MALDI-TOF) mass spectrometer, with α-cyano-4-hydroxycinnamic acid (CHCA) employed as the matrix substance. Gel-permeation chromatography (GPC) measurements were performed at 25 °C on a Waters 515 equipped with a Wyatt Technology Optilab rEX differential refractive index detector by using tetrahydrofuran as an eluent at a flow rate of 1.0 mL min^{-1}. Relative weight average (M_w) and number average (M_n) molecular weights were calculated with a calibration curve based on a group of polystyrene (PS) standard samples. Differential scanning calorimetry (DSC) thermograms were recorded on a Netzsch DSC 214 system equipped with a cooling accessory and under nitrogen atmosphere at a flow rate of 20 mL min^{-1}. Typically, about 5 mg of the solid sample was encapsulated in a sealed aluminum pan with an identical empty pan as the reference with a heating or cooling rate of 10 °C min^{-1}. Polarized optical microscopy (POM) was performed with a Nikon E400POL microscope equipped with an Instec HCS302 hot and cold stage. X-ray scattering experiments were performed at the beamline BL16B1 at the Shanghai Synchrotron Radiation Facility (SSRF) or with an Anton Paar SAXSpoint 2.0 small-angle X-ray scattering instrument. The scattering vector q is defined as $4\pi\sin\theta/\lambda$, where the wavelength λ is 0.1542 nm of Cu-K_α radiation and 2θ is the scattering angle. SANS experiments were performed using the Very Small Angle Neutron Scattering (VSANS) instrument BL-14 at the China Spallation Neutron Source (CSNS). Neutron data were collected with a wavelength range from 2.2 Å to 6.7 Å, employing a collimation length of 12.75 m. Ultraviolet visible (UV-vis) absorption spectra were recorded on a Shimadzu UV-2550 spectrometer. Circular dichroism (CD) spectroscopy measurements were carried out in a Jasco J-815 spectropolarimeter. Fluorescence spectra, absolute quantum yield and time-resolved fluorescence lifetime experiments were recorded with an Edinburgh FLS1000 transient steady-state

fluorescence spectrometer, where the time-resolved lifetime was obtained by adopting a 340 nm picosecond pulsed excitation source.

Theoretical Investigation. The ground state geometries were optimized using density functional theory (DFT), and the excited states were calculated with linear-response time-dependent DFT at the optimized ground state geometry. All calculations were performed using the Gaussian 16 package (Revision C.01) with the B3LYP hybrid functional and the 6-311G(d) basis set [1]. Grimme's D3BJ dispersion correction was applied to enhance the accuracy of the calculations [2].

The conformational space of the long-chain polymer was explored using the Conformer-Rotamer Ensemble Sampling Tool (CREST) implemented in the xtb-6.3.3 package. Geometry optimizations were performed in the gas phase at the semiempirical GFN2-xTB level, as implemented in the xtb, to obtain the most stable conformers [3]. The GFN2-xTB method is derived from a Hamiltonian similar to DFTB3, employs a minimal valence basis set centered on atoms (STO-mG), and incorporates the density-dependent D4 dispersion correction.

The Col_{ho} phase was modeled using the program Accelrys Materials Studio. The modeling was performed in a *P*1 unit cell without symmetry constraints. The parameters for the columnar structure determined by SAXS are $a = b = 2.76$ nm and $c = 1.34$ nm, with four molecules per columnar stratum. The mesogenic units were rotated by 30° relative to each other along the column axis. After constructing the unit cell, geometry optimization was performed using the Forcite module with the COMPASSII force field and an atom-based summation method. Final structural refinement was carried out using Pawley-type refinement.

Synthetic Protocols

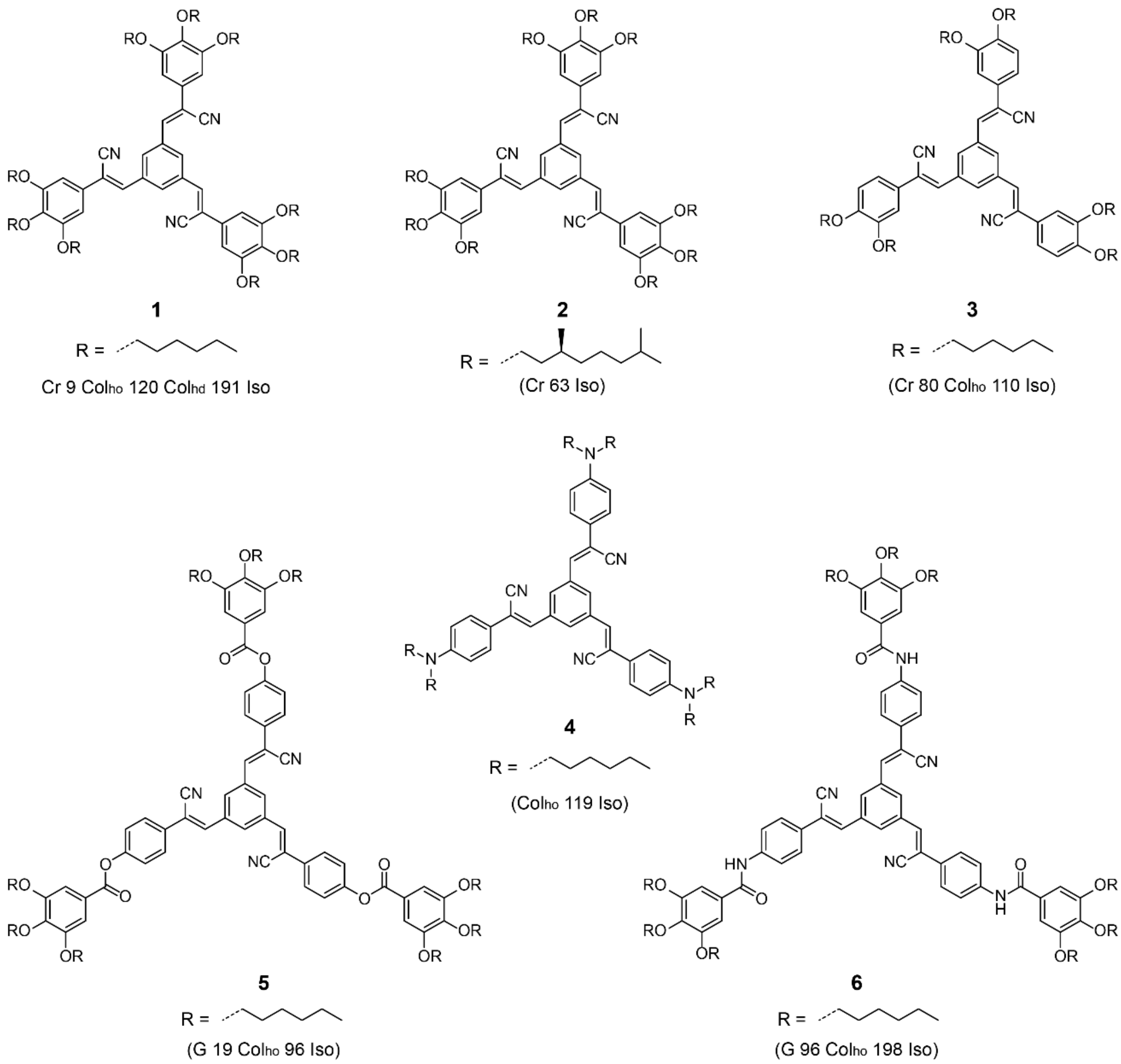

Figure S1. Summary of discotic LC monomers and their phase behaviors. Chemical structures and their thermotropic phase transition behaviors of discotic LC monomers **1-6** used in this work. Abbreviations: Cr, crystalline; G, glassy; Col_{ho}, ordered hexagonal columnar LC; Col_{hd}, disordered hexagonal columnar LC; Iso, isotropic. Among the six CS-based discotic monomers, **3**-**6** were synthesized and characterized following our previously reported procedures [4–6]. Monomers **1** and **2** were synthesized using analogous methods; the synthesis of **1** is described herein as a representative example.

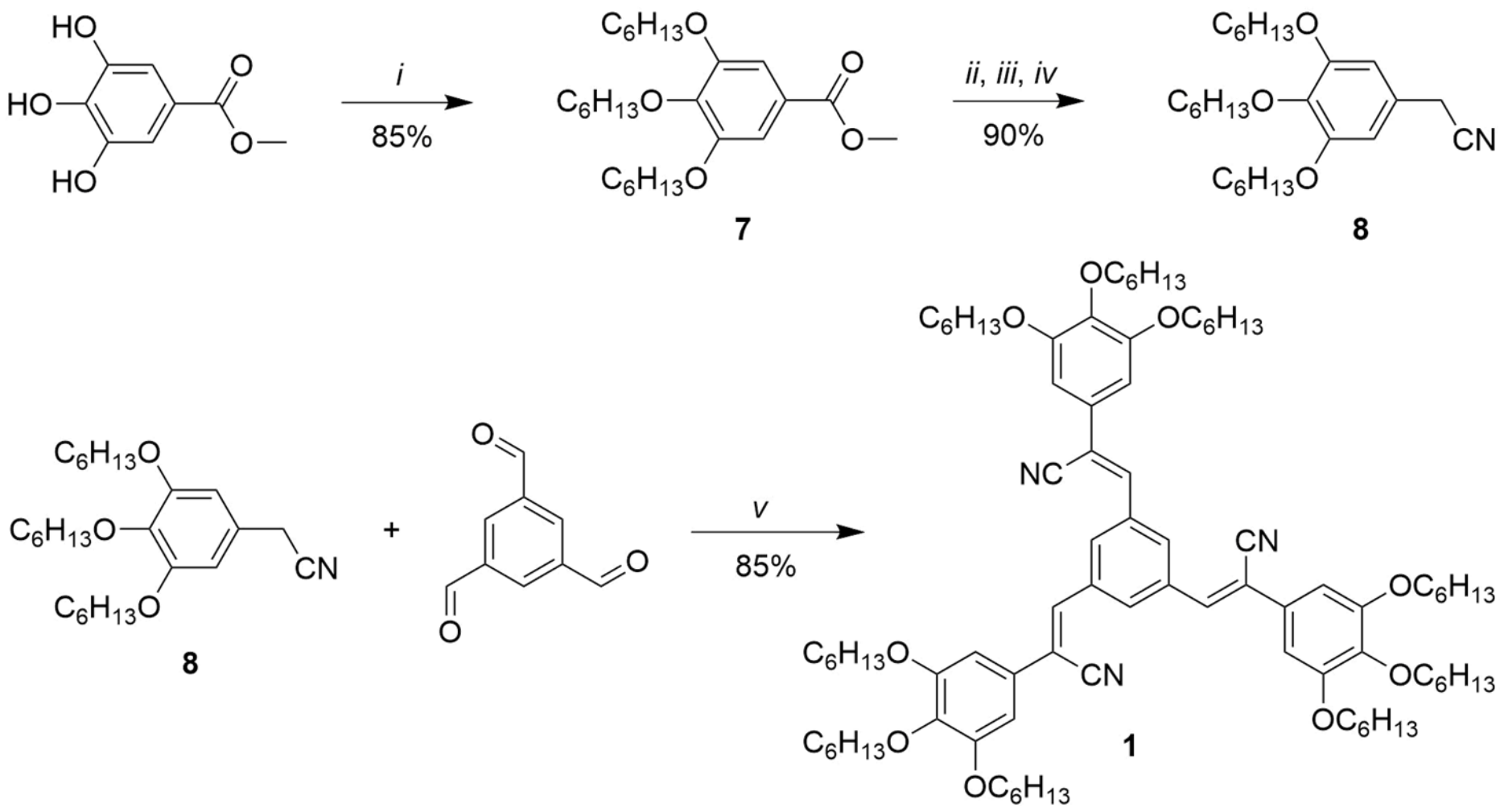

Figure S2. Synthesis of monomer **1**. Reagents and conditions: (i) 1-bromohexane, tetrabutylammonium iodide, potassium carbonate, *N,N*-dimethylformamide, 80°C, 12 h; (ii) lithium aluminium hydride, tetrahydrofuran, 0~25°C, 2.5 h; (iii) phosphorus tribromide, dichloromethane, 0~25°C, 5 h; (iv) trimethylsilyl cyanide, tetrabutylammonium fluoride, acetonitrile, 25°C, 2.5 h; (v) tetrabutylammonium hydroxide, methanol, 55°C, 2 h.

Methyl 3,4,5-tris(hexyloxy)benzoate (7). A mixture of methyl gallate (1.8 g, 9.8 mmol), 1-bromohexane (6.4 g, 39 mmol), tetrabutylammonium iodide (0.1 g, 0.3 mmol), and potassium carbonate (11 g, 77 mmol) in 100 mL of *N,N*-dimethylformamide was stirred at 80 °C for 12 h. After cooling to room temperature, the reaction mixture was poured into water and extracted with dichloromethane. The combined organic layers were concentrated, and the crude product was purified by silica-gel column chromatography using mixed solvents of petroleum ether/ethyl acetate (20:1, v/v) as the eluent to give a desired product (3.8 g) in white solid. Yield 90%. ^{1}H NMR (400 MHz, $CDCl_3$): δ (ppm) = 7.25 (s, 2H), 4.05-3.97 (m, 6H), 3.89 (s, 3H), 1.86-1.70 (m, 6H), 1.52-1.19 (m, 18H), 0.88 (t, 9H, J = 6.4 Hz).

2-(3,4,5-tris(hexyloxy)phenyl)acetonitrile (8). To a solution of **7** (1.2 g, 2.7 mmol) in 40 mL tetrahydrofuran, lithium aluminium hydride in tetrahydrofuran (1.0 mol L^{-1}, 4.0 mL) was added dropwise at 0°C over 30 minutes. The reaction mixture was then stirred at room temperature for 2 h. Upon completion, the reaction was quenched with water, and the mixture was extracted with dichloromethane. The organic phase was dried over anhydrous magnesium sulfate, filtered, and

concentrated under reduced pressure to afford (3,4,5-tris(hexyloxy)phenyl)methanol in white solid.

The obtained alcohol was dissolved in 15 mL dichloromethane, and a solution of phosphorus tribromide (0.9 g, 3.4 mmol) in dichloromethane was added dropwise at 0°C over 2 h. The reaction mixture was stirred at room temperature for 3 h. The reaction was quenched with water, and the resulting mixture was extracted with dichloromethane. The obtained organic layers were dried over anhydrous magnesium sulfate, filtered, and concentrated under reduced pressure to afford 5-(bromomethyl)-1,2,3-tris(hexyloxy)benzene in white solid.

A mixture of the obtained bromide, trimethylsilyl cyanide (0.3 mL, 3.8 mmol), and tetrabutylammonium fluoride (1.0 g, 4.0 mmol) in 30 mL acetonitrile was stirred at room temperature for 2.5 h. After the reaction was complete, the solvent was removed under reduced pressure. The residue was dissolved in dichloromethane and washed with water. The crude product was purified by neutral aluminum oxide column chromatography using dichloromethane as the eluent to give a desired product (1.0 g) in white solid. Yield 90%. ^{1}H NMR (400 MHz, $CDCl_3$): δ (ppm) = 6.48 (s, 2H), 4.00-3.90 (m, 6H), 3.66 (s, 2H), 1.84-1.69 (m, 6H), 1.52-1.27 (m, 18H), 0.90 (t, 9H, J = 6.5 Hz).

(2Z,2'Z,2''Z)-3,3',3''-(benzene-1,3,5-triyl)tris(2-(3,4,5-tris(hexyloxy)phenyl)acrylonitrile) (1). A solution of **8** (0.8 g, 1.9 mmol) and benzene-1,3,5-tricarbaldehyde (0.09 g, 0.58 mmol) in 20 mL ethanol was treated with tetrabutylammonium hydroxide (0.6 mL, 40% in methanol) and stirred at 55°C for 2 h. After cooling to room temperature, the resulting precipitate was collected by filtration. The crude product was purified by silica-gel column chromatography using mixed solvents of petroleum ether/ethyl acetate (8:1, v/v) as the eluent to give a desired product (0.67 g) in light yellow solid. Yield 85%. ^{1}H NMR (400 MHz, $CDCl_3$): δ (ppm) = 8.28 (s, 3H), 7.52 (s, 3H), 6.88 (s, 6H), 4.12-3.98 (m, 18H), 1.90-1.72 (m, 18H), 1.58-1.28 (m, 54H), 0.91 (t, 27H, J = 6.7 Hz). ^{13}C NMR (100 MHz, $CDCl_3$): δ (ppm) = 152.53, 138.83, 138.22, 134.36, 129.40, 127.76, 116.58, 113.45, 103.92, 72.62, 68.41, 30.73, 30.55, 29.25, 28.31, 24.74, 24.72, 21.67, 21.61, 13.08, 13.01. MS (m/z): $[M + H]^+$ calcd 1360.9807, found 1360.9787; $[M + Na]^+$ calcd 1382.9627, found 1382.9624; $[M + K]^+$ calcd 1398.9366, found 1398.9369.

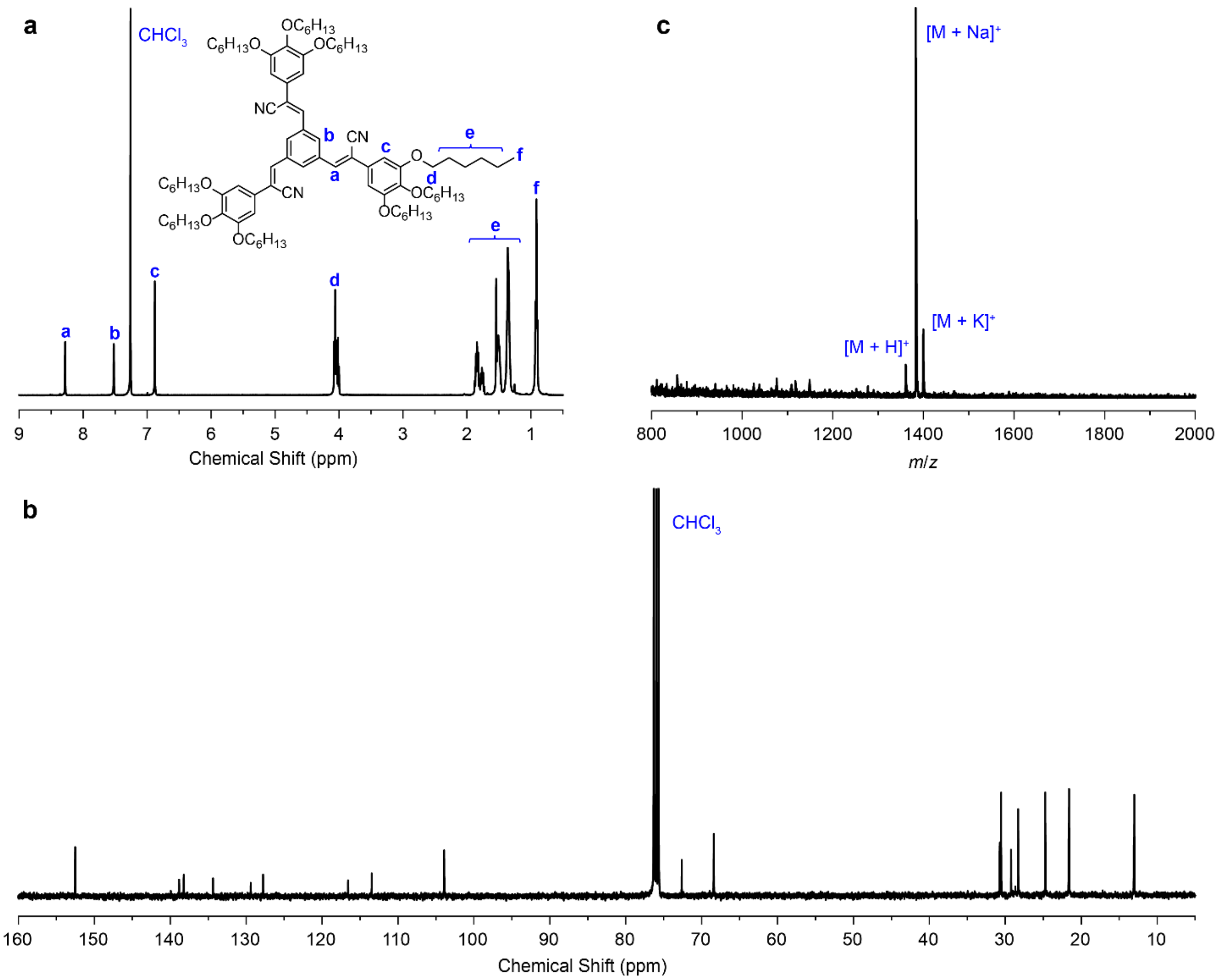

Figure S3. Molecular characterization of **1**. (a) ^{1}H NMR spectrum, (b) ^{13}C NMR spectrum, and (c) mass spectrum of **1** used in this work.

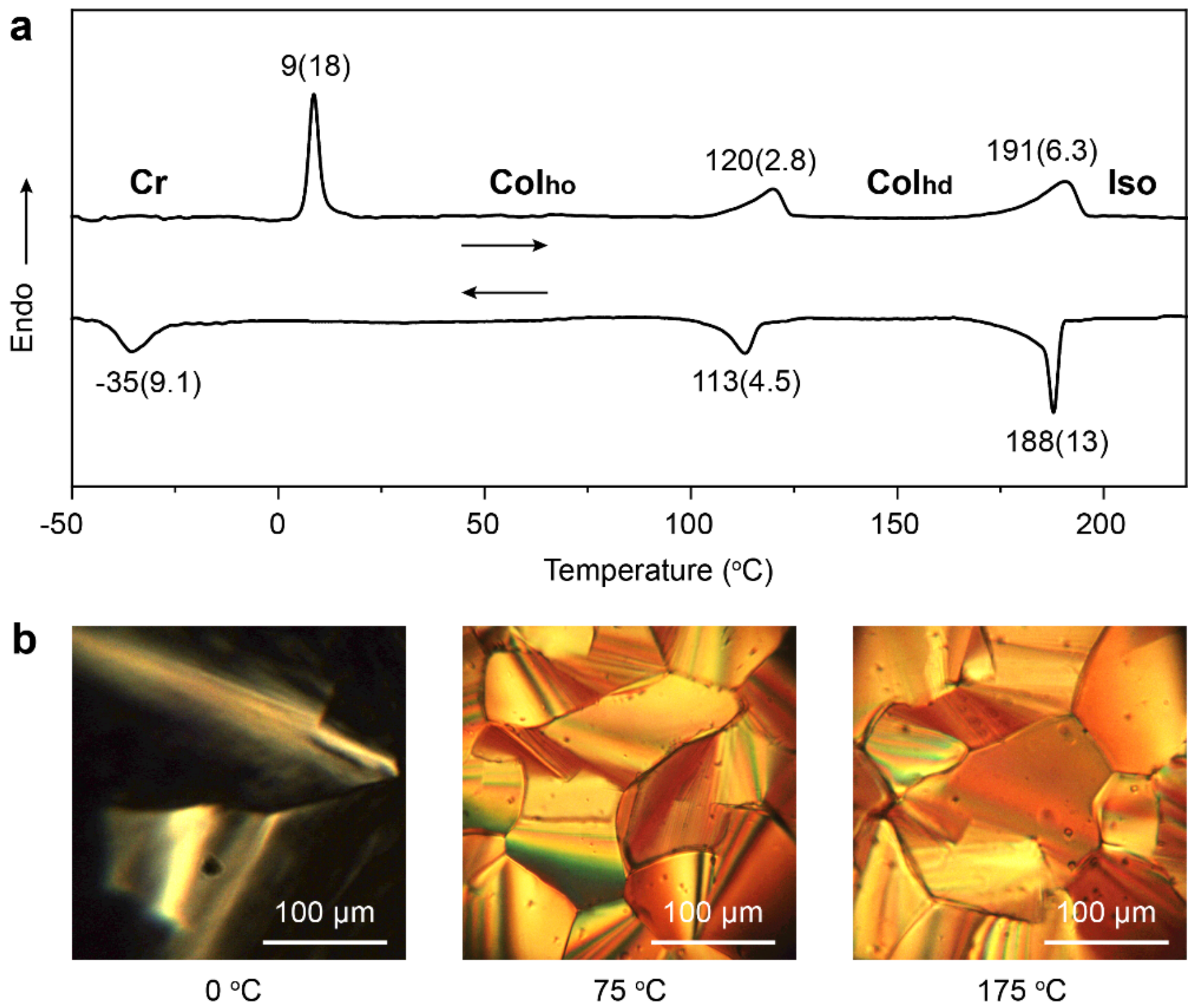

Figure S4. Thermotropic LC behavior of **1**. (a) DSC thermograms recorded at a scanning rate of 2 °C min^{-1}, indicated with transition temperature (in °C) and associated enthalpy changes (in parentheses, in kJ mol^{-1}). (b) POM images under crossed polarizers at indicated temperatures after cooling from Iso liquid.

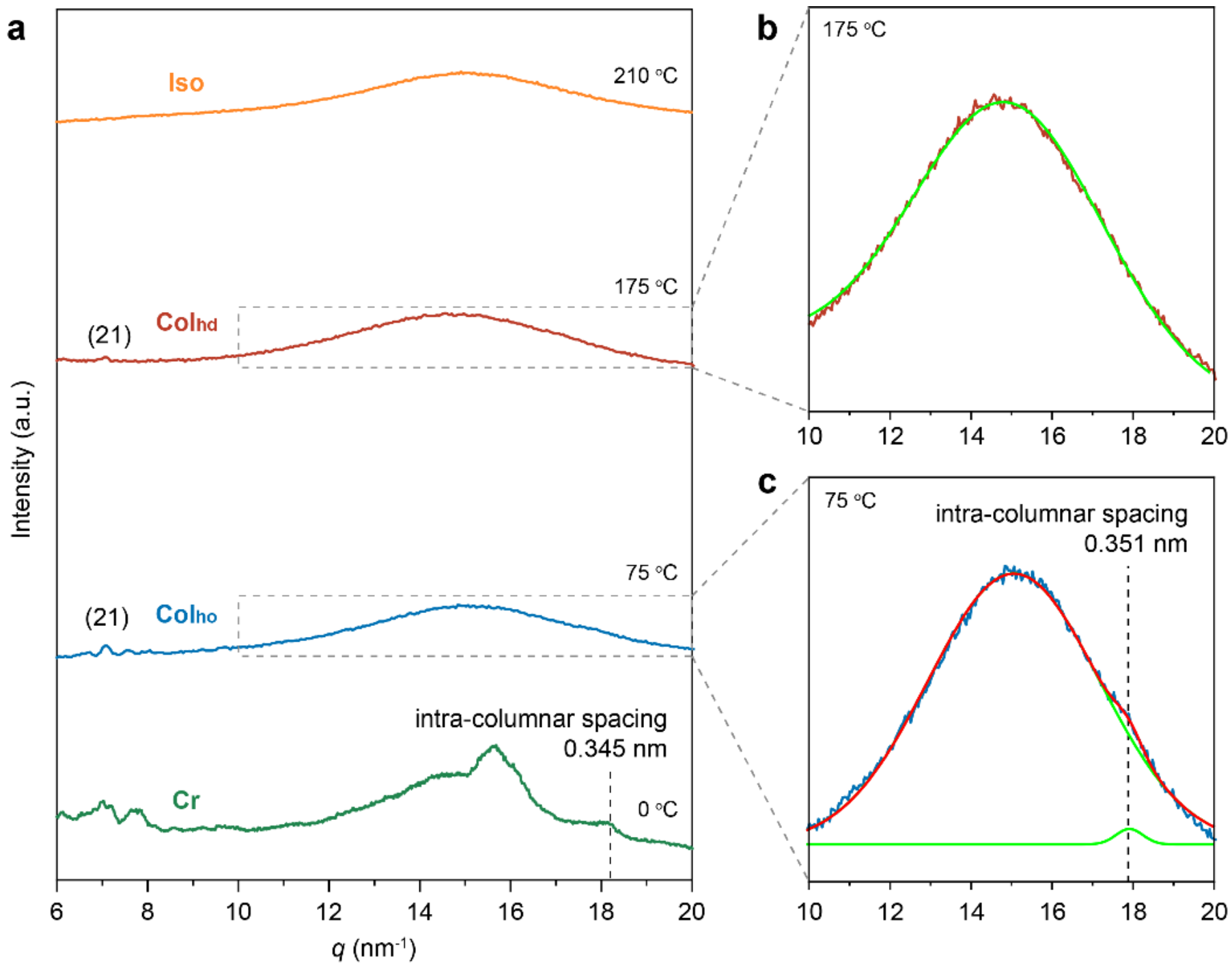

Figure S5. Temperature-dependent WAXS of **1**. (a) WAXS profiles with proposed indexing during heating process. (b) Enlarged WAXS profile at 175°C, showing obvious melting aliphatic halo. (c) Enlarged WAXS profile at 75°C, showing clear intracolumnar order with an average intermolecular spacing of 0.351 nm.

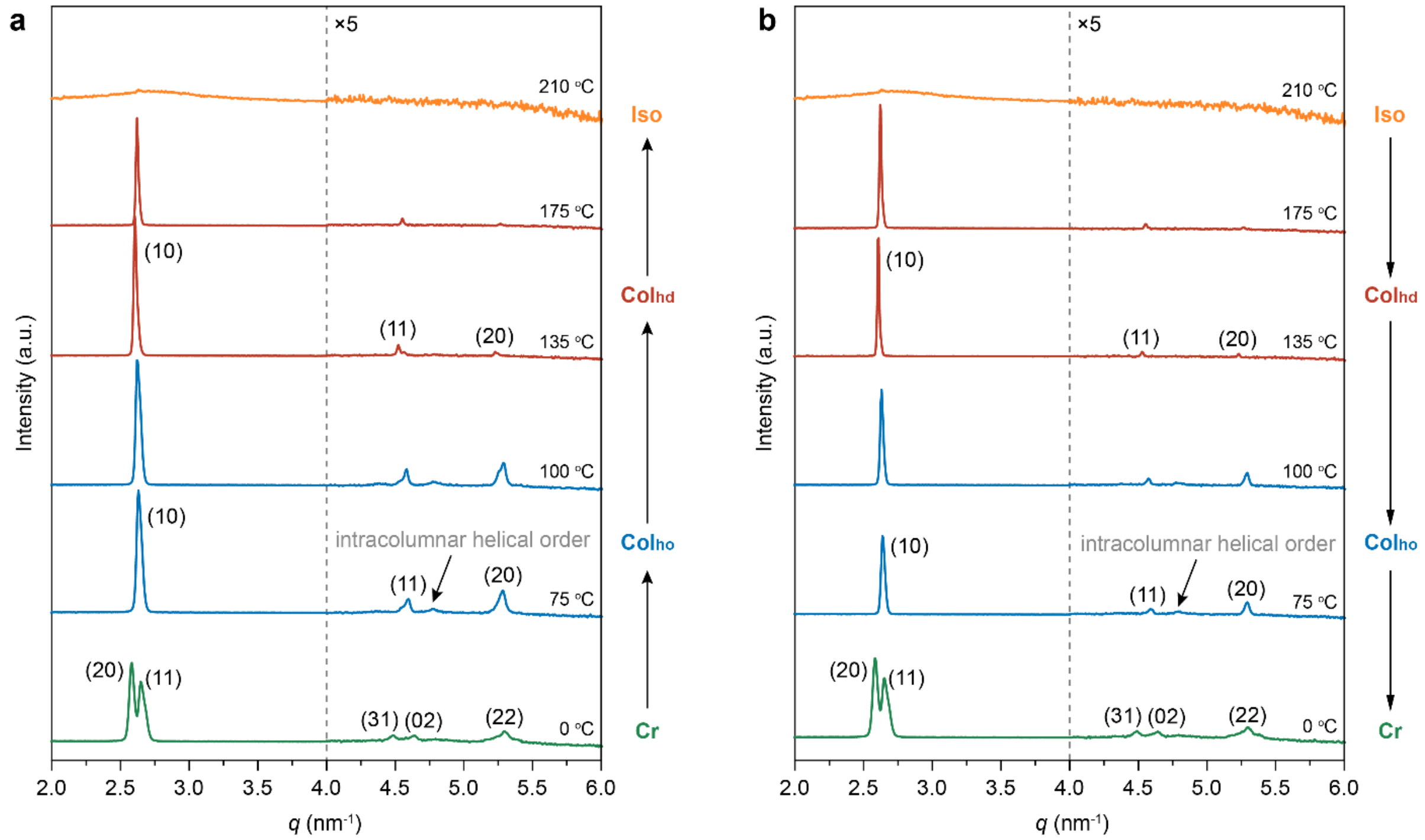

Figure S6. Temperature-dependent SAXS of **1**. (a) SAXS profiles with proposed indexing during heating process. (b) SAXS profiles with proposed indexing during cooling process.

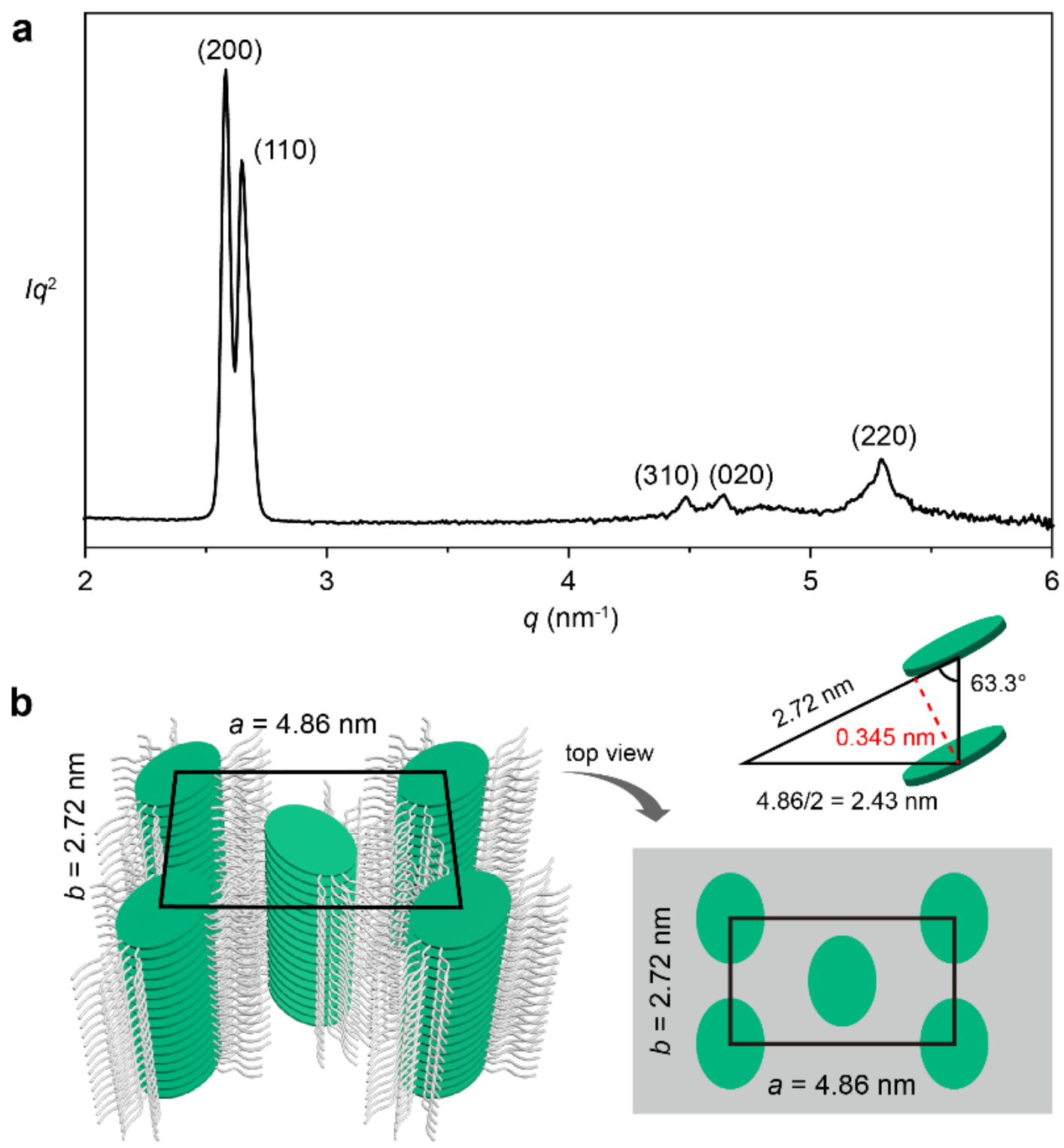

Figure S7. Molecular packing arrangement of **1** in Cr phase. (a) SAXS profile with proposed indexing at 0°C. (b) Schematic illustration of the proposed columnar stacking with two-dimensional rectangular symmetry in the crystal phase (Cr).

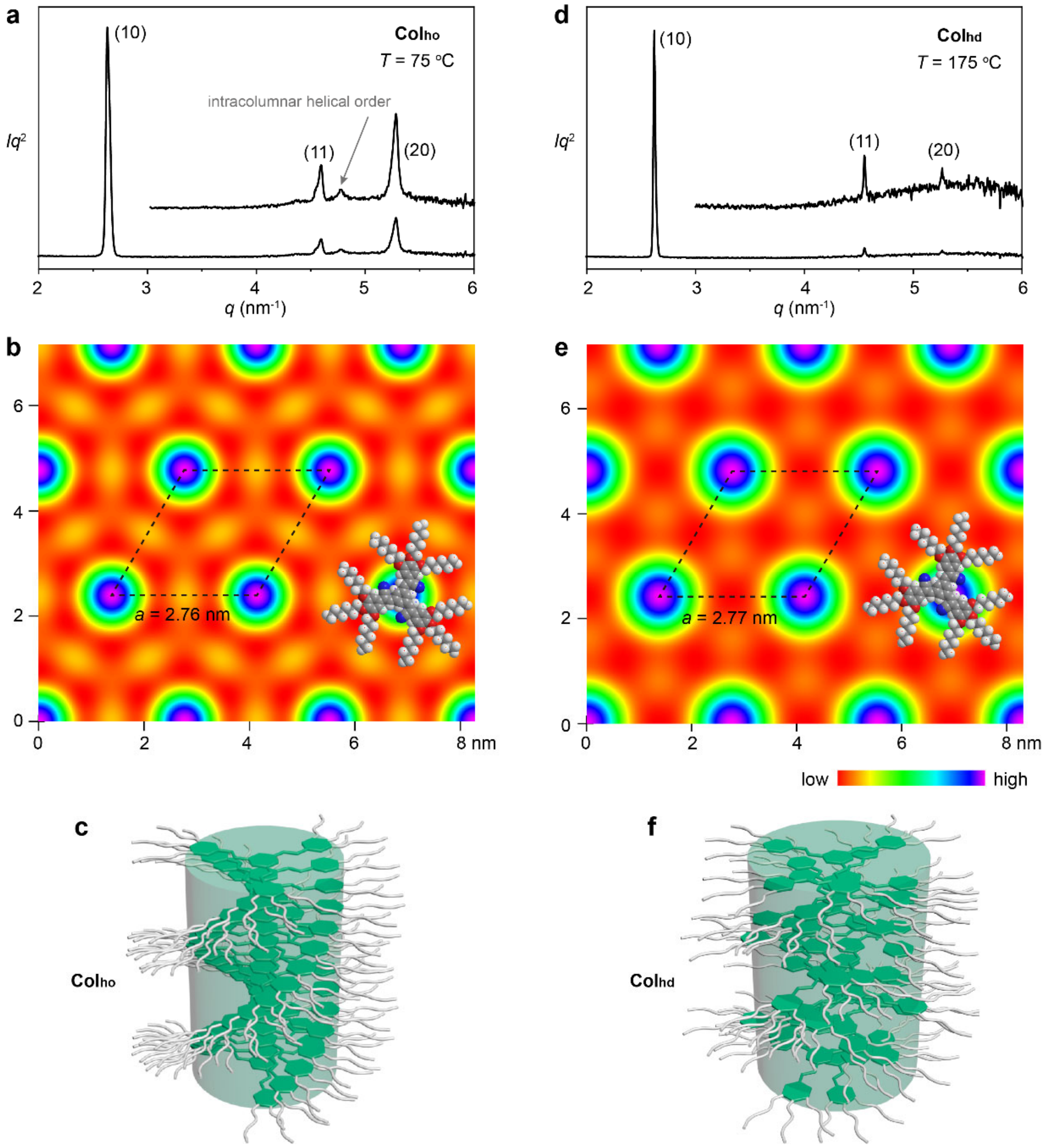

Figure S8. Proposed molecular packing arrangements of **1** in LC phases. (a) SAXS profile at 75°C with proposed indexing. The broad intensity at 4.77 nm^{-1} is attributed to the intracolumnar helical packing with a helical pitch and repeat of 3.96 nm, which indicates a helical angel of ca. 30° due to the C_3-symmetrical CS mesogen. (b) Reconstructed electron density map from the SAXS intensities, showing comparable high electron density region with the dimension of the aromatic CS core. (c) Schematic illustration of the proposed intracolumnar ordered helical packing in the Col_{ho} phase. (d) SAXS profile at 175°C with proposed indexing. (e) Reconstructed electron density map from the SAXS intensities, showing relatively larger high electron density region compared to the dimension of the aromatic CS core. (f) Schematic illustration of the proposed intracolumnar disordered packing in the Col_{hd} phase.

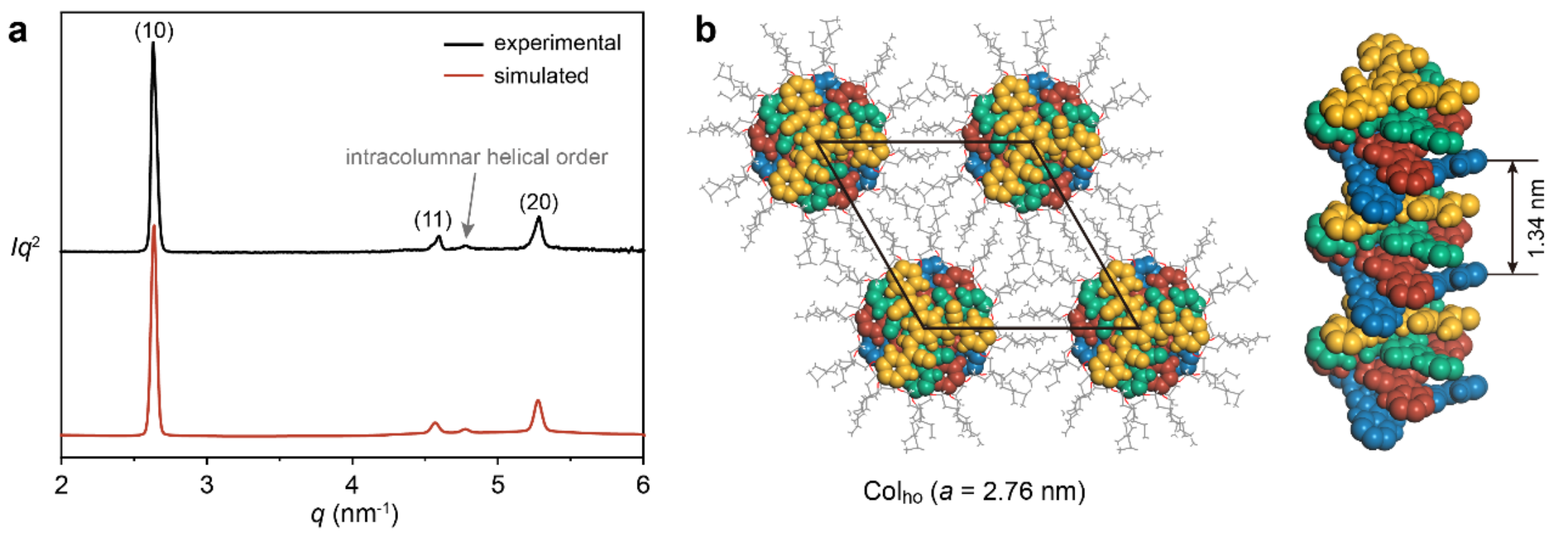

Figure S9. Structural simulation of the Col$_{ho}$ structure of **1**. (a) SAXS profiles of **1** at 75°C in Col$_{ho}$ phase with proposed indexing. The broad intensity at 4.7 nm^{-1} is attributed to the helical packing. (b) Model of the Col$_{ho}$ structure of **1**. The π-conjugated aromatic cores and alkyl chains are shown with space-filling and stick models, respectively. The black rhombus represents the unit cell.

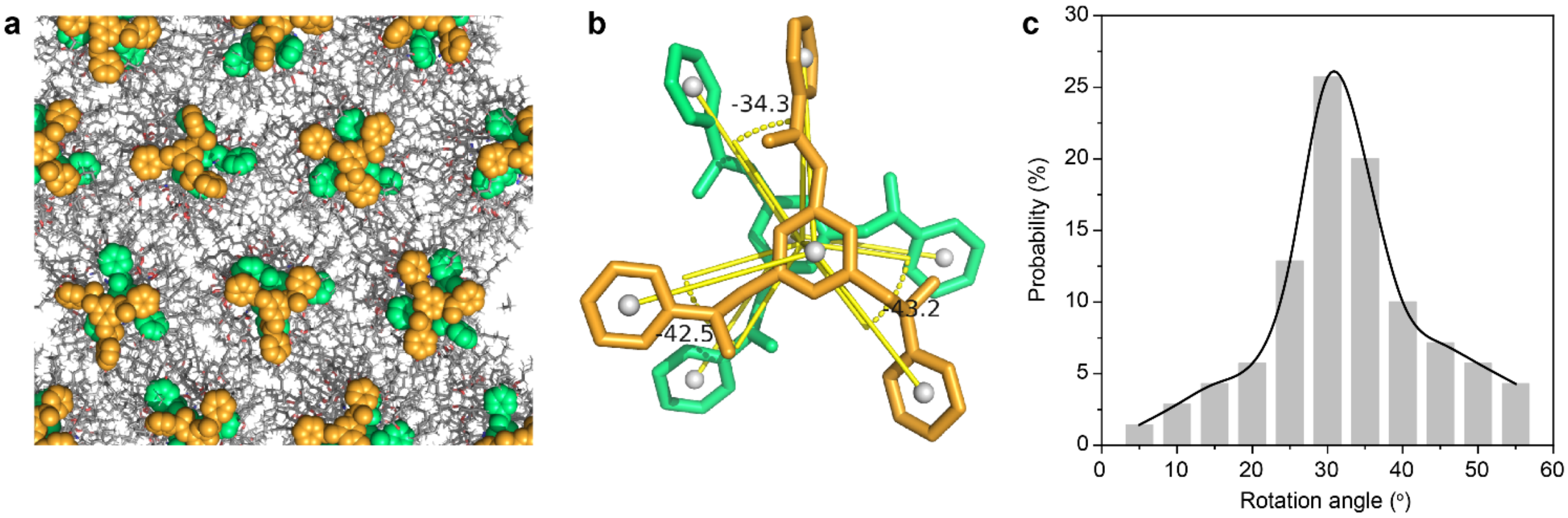

Figure S10. Molecular dynamics simulations of the Col$_{ho}$ structure of **1**. (a) Representation of the hexagonal columnar structure of **1** using molecular dynamics simulations. The π-conjugated aromatic cores and alkyl chains are shown with space-filling and stick models, respectively. (b) Schematic illustration of the method used to measure the rotation angle between neighboring molecules. (c) Distribution of the relative orientations between the neighboring molecules for **1**, derived from molecular dynamics simulations. The probability reflects the likelihood of two neighboring molecules being rotated with respect to one another.

Thermotropic Phase Behaviors of 1. The phase transition behaviors of **1** were investigated using differential scanning calorimetry (DSC), polarized optical microscopy (POM), and small- and wide-angle X-ray scattering (SAXS and WAXS). Monomer **1** exhibits three distinct thermal transitions in heating DSC thermogram (Fig. S4a), corresponding to sequential phase transitions from a columnar crystalline (Cr) phase to an ordered hexagonal columnar LC (Col_{ho}) at 9°C, followed by a transition to a disordered hexagonal columnar LC (Col_{hd}) above 120°C, and finally to an isotropic (Iso) liquid phase above 191°C. These phase transitions are obviously distinguishable under POM, as evident from the representative texture characteristic of columnar phases at various temperatures (Fig. S4b). Phase assignments were further validated using temperature-dependent WAXS (Fig. S5) and SAXS (Fig. S6).

WAXS profiles at lower temperatures exhibit well-defined diffraction peaks in the wide-angle region, indicative of the Cr phase. Upon heating, these peaks evolve into broad halos, confirming the transition to LC states (Fig. S5). Notably, at 75°C, intracolumnar π-stacking is observed, with an average intermolecular spacing of 0.351 nm. This π-stacking signal vanishes at 175°C, manifesting a transition from the ordered columnar phase to the disordered columnar phase.

SAXS measurements provided complementary insights into intercolumnar organization (Fig. S6). At 0°C, the SAXS profile reveals five distinct scattering peaks, including two intense peaks at 2.58 and 2.65 nm^{-1}, supporting the assignment of a rectangular columnar crystalline (Cr) phase (Fig. S7a). In this phase, the discotic CS molecules are tilted by approximately 63.3° relative to the columnar axis (Fig. S7b), which correlates with the reduced intracolumnar spacing of approximately 0.345 nm observed by WAXS (Fig. S5). The assignment of Cr phase closely resembles that of conventional discotic LCs composed of polycyclic aromatic mesogens [7].

At 75°C and 175°C, SAXS profiles display a dominant primary peak accompanied by two weaker peaks of similar half-width, corresponding to the (10), (11), and (20) reflections of a hexagonal positional order characteristic of columnar LC phases (Fig. S8a,d). The calculated unit cell parameters (a = 2.76 nm or 2.77 nm) align well with the molecular dimension. A distinguishing feature between the Col_{ho} and Col_{hd} phases is the presence of a broad peak at 4.7 nm^{-1} in the Col_{ho} phase, corresponding to a periodicity along the columnar axis equal to four molecules, indicative of a helical molecular arrangement within the columns.

Further differentiation between the Col_{ho} and Col_{hd} phases is revealed by reconstructing electron density maps. In both phases, electron densities are highest at the center of the columns and lower at

the periphery, suggesting central localization of the aromatic cores. However, in the Col_{ho} phase, the electron density distribution aligns well with the molecular dimension (Fig. S8b), supporting a helical intracolumnar packing of three-armed discotic molecules (Fig. S8c). In contrast, the Col_{hd} phase displays a broader electron-rich region (Fig. S8e), consistent with the disordered aromatic core packing (Fig. S8f), in agreement with the absence of π-stacking signal in WAXS profile. These results indicate that the arrangement of the molecules inside the columns significantly changes by the Col_{ho}-Col_{hd} phase transition.

To gain further insight into the Col_{ho} structure, we constructed a molecular model using Accelrys Materials Studio (Fig. S9). In this model, neighboring molecules of **1** within a column are laterally rotated by ~30°, leading to a helical stacking with four molecules per pitch. To further validate this model, molecular dynamics simulations were conducted to examine the columnar arrangement of **1** at the microscopic molecular level (Fig. S10). After equilibration, we observe that the molecules were stacked helically within the columns, with twist angles clustering around 30°, and the columns arranged in a hexagonal lattice. The combination of experiment results and theoretical simulations substantiate the helical columnar model of such three-armed discotic CS molecules.

Light-Activated TCP Methods. Monomers (~10 mg) were thermally pressed onto a quartz substrate (5 × 5 cm) at a temperature above its clearing point, then cooled to specified temperature (e.g. 75°C) to form a thin film. The film was annealed at this temperature for 10 min and then placed under a 365 nm UV lamp until the film became colorless and nearly transparent, yielding the polymer product. The resulting polymer was either peeled from the substrate as a solid or dissolved in an appropriate solvent (e.g. $CDCl_3$, tetrahydrofuran) prior to characterization.

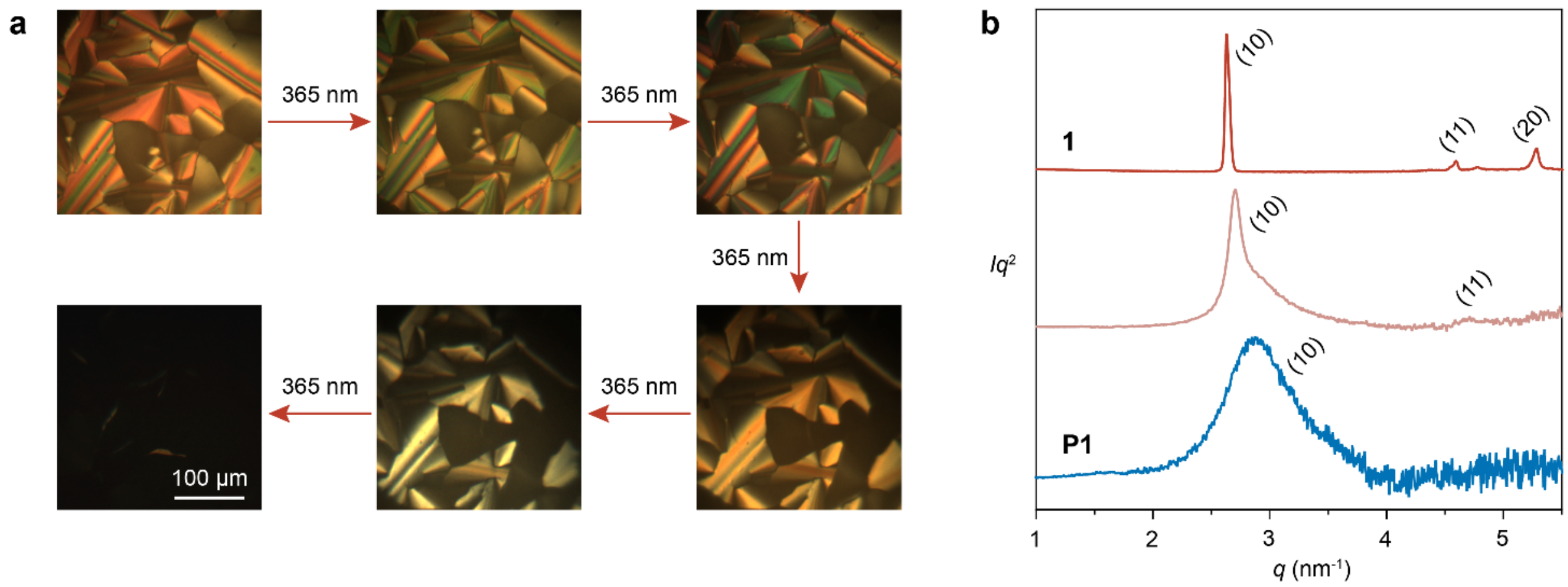

Figure S11. Structural evolution of **1** during TCP at 75°C in Col_{ho} phase. (a) POM images under 365 nm light irradiation, showing a gradual loss of columnar LC order during TCP. (b) SAXS profiles illustrating a transition from a highly ordered Col_{ho} structure to a weakly ordered columnar phase, accompanied with a reduction in columnar dimensions.

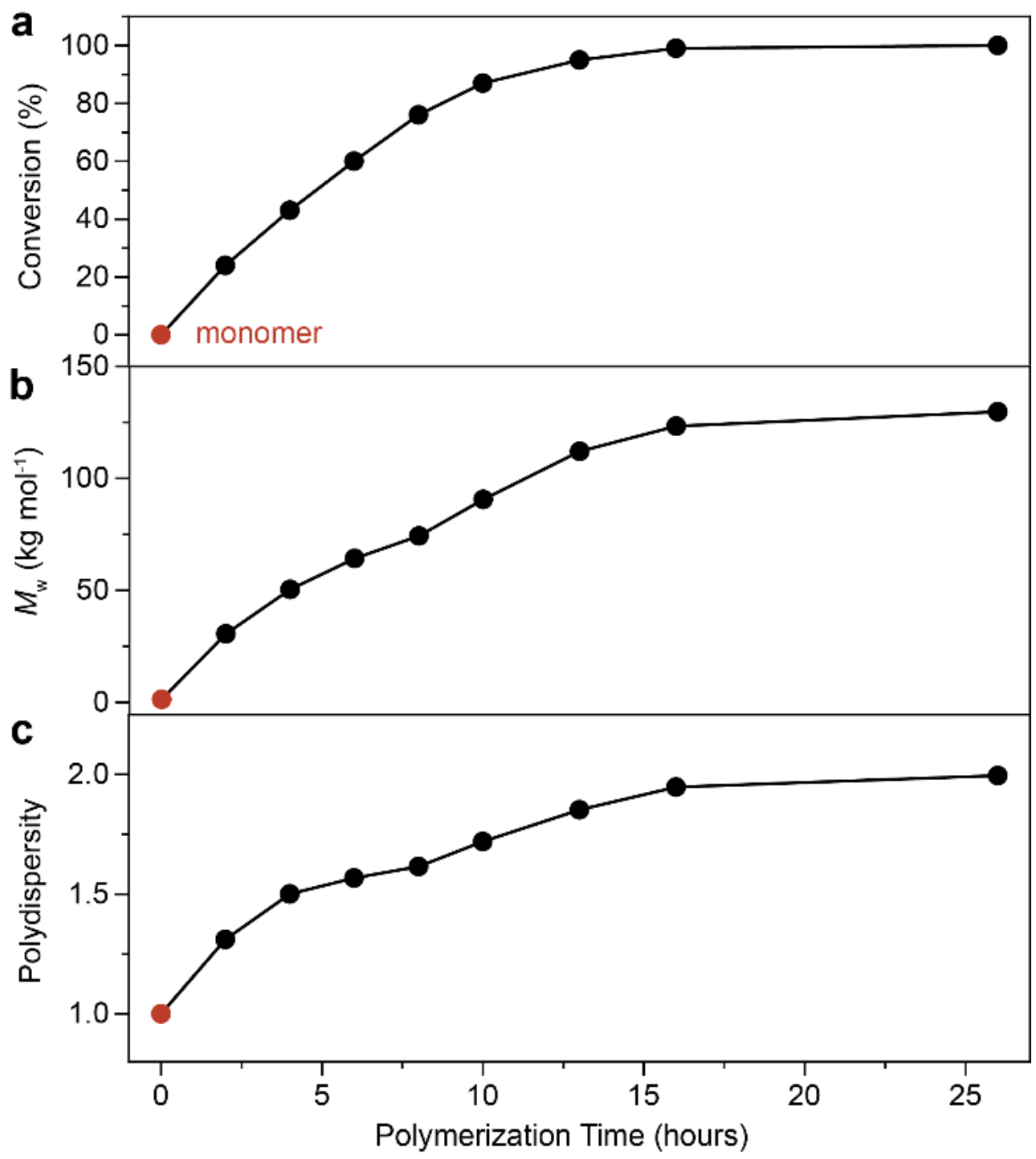

Figure S12. Light-activated TCP parameters of **1** at 75°C. (a) Monomer conversion, (b) weight-average molecular weight (M_w), and (c) polydispersity index (PDI) as determined by GPC analysis of the obtained polymers through TCP at 75°C with various polymerization times. The red point indicates the monomer **1**.

Table S1. Summary of the polymerization parameters for the light-activated TCP of **1** at 75°C.

Time (hours)	0	2	4	6	8	10	13	16	26
Conversion (%)	0	24	43	60	76	87	95	99	99.9
M_w (kg mol^{-1})	1.7	30.7	50.4	64.3	74.3	90.6	112.1	123.3	129.7
PDI	1.04	1.31	1.50	1.57	1.62	1.72	1.85	1.95	1.99

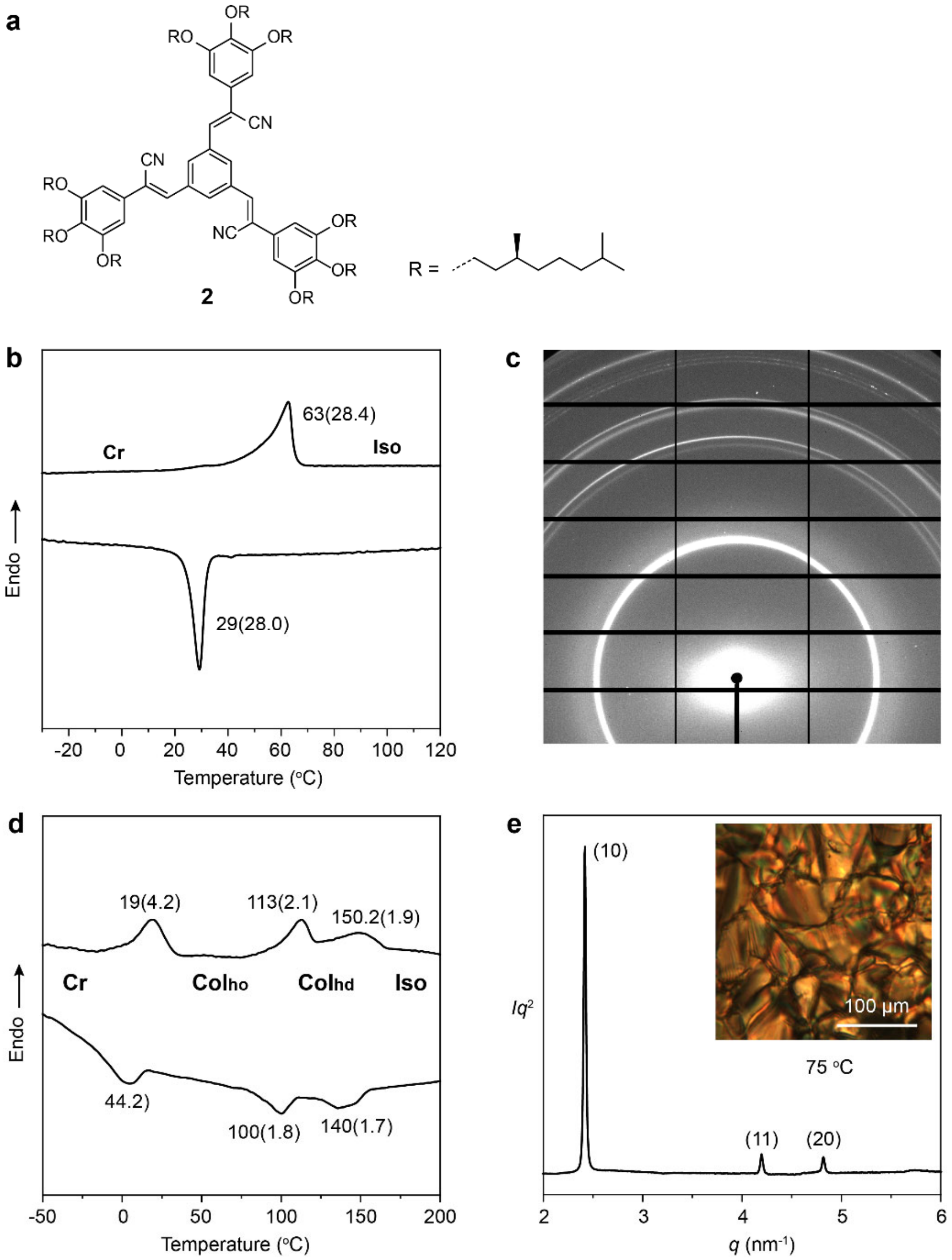

Figure S13. Thermotropic LC behavior of complex **1/2**. (a) Chemical structure of **2**, featuring nine chiral alkoxy chains. (b) DSC thermograms of **2** recorded at a scanning rate of 10 °C min^{-1}, showing no LC transition. Transition temperature (in °C) and associated enthalpy changes (in parentheses, in kJ mol^{-1}) are indicated. (c) The SAXS pattern of **2** in the crystal phase at room temperature. (d) DSC thermograms of complex **1/2** (5/1 molar ratio) measured at a scanning rate of 10 °C min^{-1}, displaying phase transitions similar to those of pure **1**. Transition temperature (in °C) and associated enthalpy changes (in parentheses, in kJ mol^{-1}) are indicated. (e) SAXS profile of complex **1/2** (5/1) at 75 °C in the Col$_{ho}$ phase, with the inset showing a POM image under crossed polarizers at 75 °C after cooling from Iso liquid.

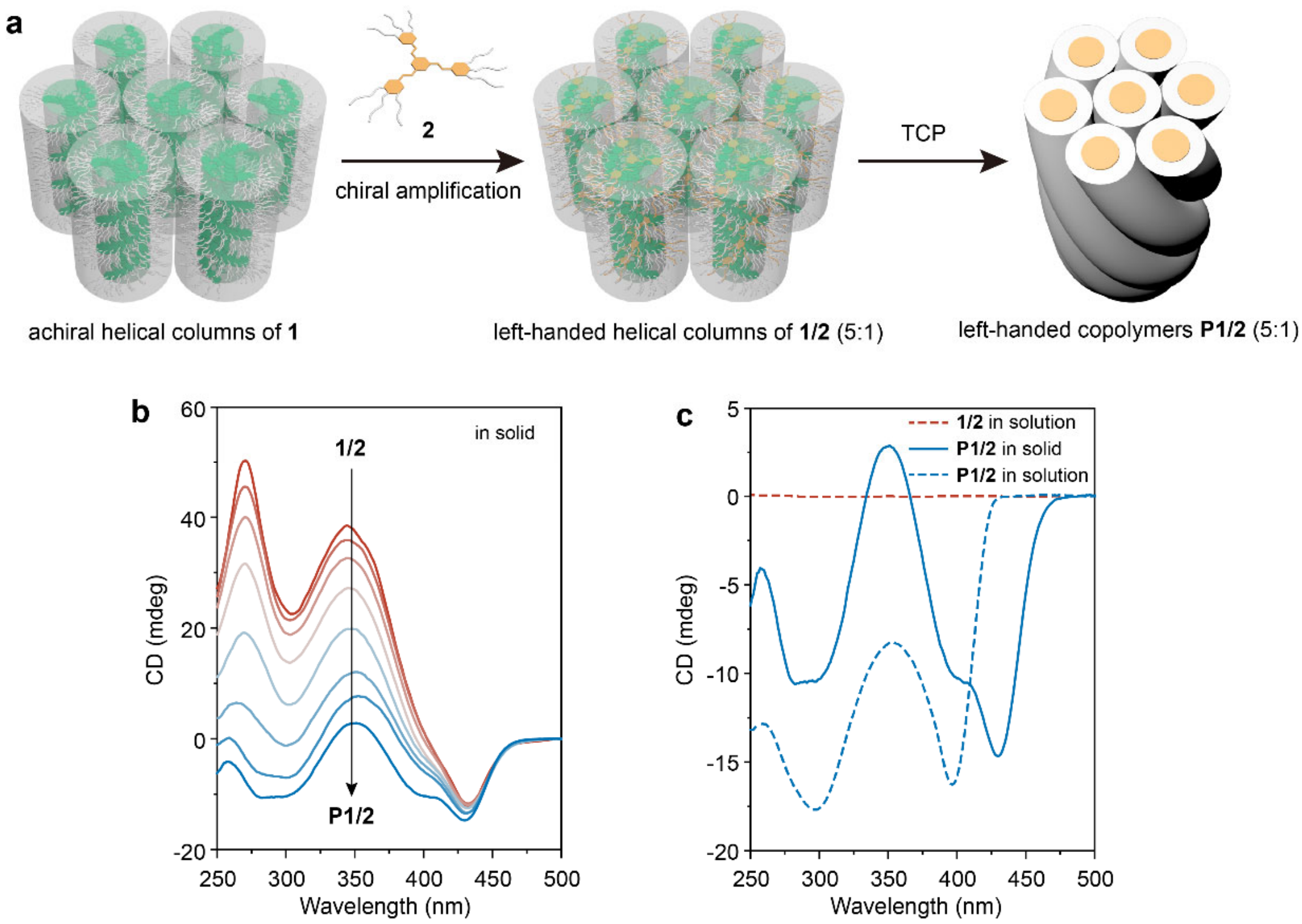

Figure S14. Induction of chirality in TCP of **1/2**. (a) Co-stacking assembly of **1** and **2**, the latter bearing chiral substituents, leads to the formation of a left-handed helical columnar LC structure, which directs the formation of left-handed helical polymers upon TCP. (b) CD spectra recorded during the copolymerization of **1** and **2** upon 365 nm light irradiation. (c) CD spectra the of complex **1/2** and the resulting copolymer **P1/2** (**1/2** = 5:1) in dichloromethane solution and in the solid state.

Helical Conformation Judged by Induced Chirality. As the chiral monomer **2** exhibits only a crystalline phase (Fig. S13a-c), it is unsuitable for TCP due to the geometrical constraints. To validate the formation of helical polymer structures following TCP, we performed a copolymerization of monomer **1** and chiral monomer **2**. The coassembly of **1** and **2** (5:1) displayed phase behavior similar to that of pure monomer **1** (Fig. S13d,e). However, significant changes in intracolumnar molecular packing were observed, attributed to chiral induction, resulting in the formation of single-handed helical columns. As a result, TCP is expected to yield polymers with defined single-handed chirality (Fig. S14a). As anticipated, the coassembled film in the Col_{ho} phase exhibited a distinct CD signal at 350 nm with a positive Cotton effect (Fig. S14b). Upon 365 nm light irradiation, the CD signal at 350 nm gradually diminished, while a negative CD signal at 430 nm gradually enhanced. Furthermore, the CD activity of the resulting polymer persisted in solution (Fig. S14c), supporting the presence of a stable helical conformation.

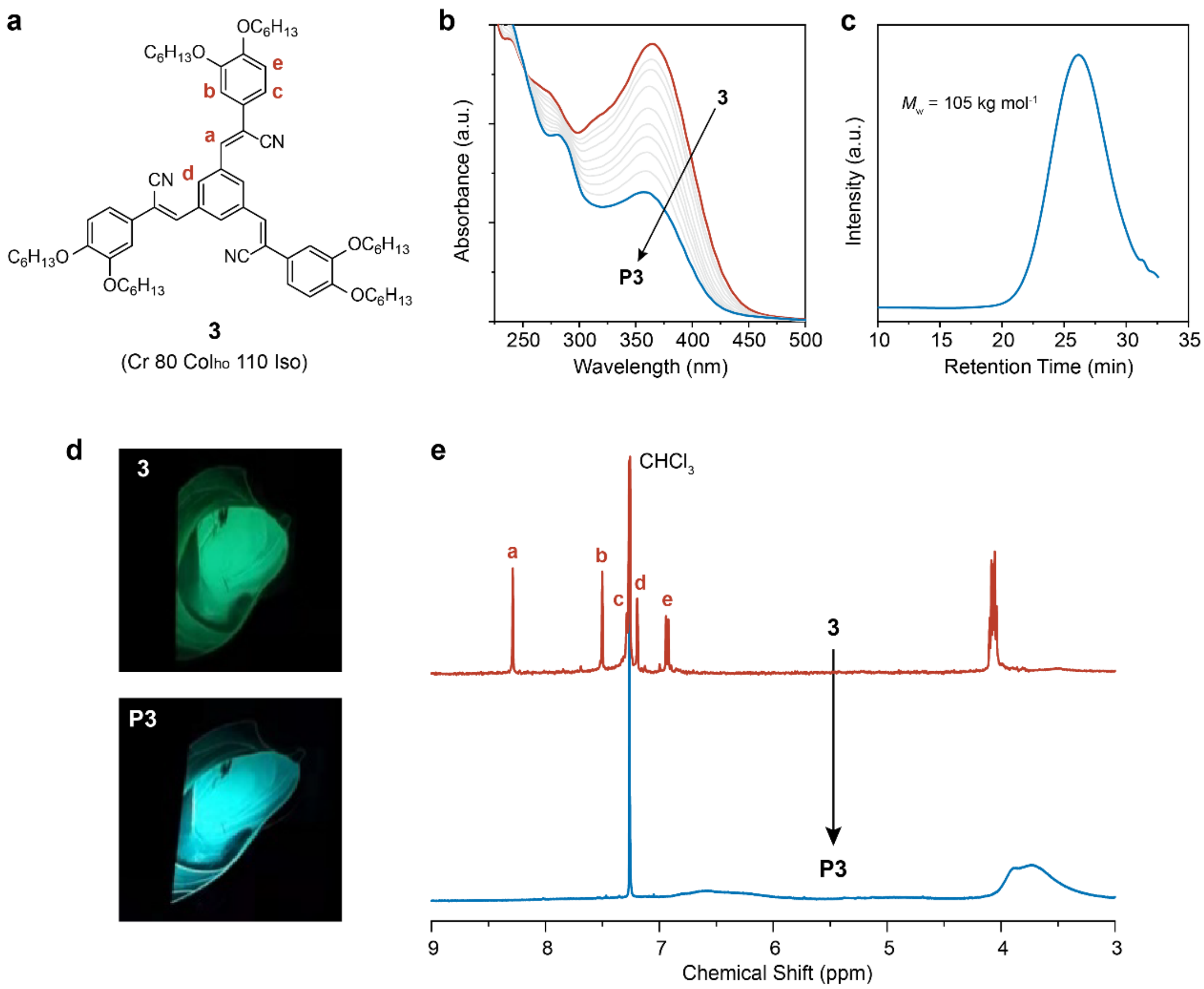

Figure S15. Light-activated TCP of **3** at 90°C in Col$_{ho}$ phase. (a) Chemical structure of **3**, featuring six peripheral alkoxy chains, along with its thermotropic phase behavior (adapted from our previous work [4]). (b) UV-vis absorption spectra of a thin film of **3** recorded under 365 nm light irradiation, demonstrating the progression of polymerization. (c) GPC trace of the resulting polymer **P3**. (d) Fluorescence images of **3** and **P3** following light-activated TCP. (e) ^{1}H NMR spectra of **3** and **P3** in $CDCl_3$.

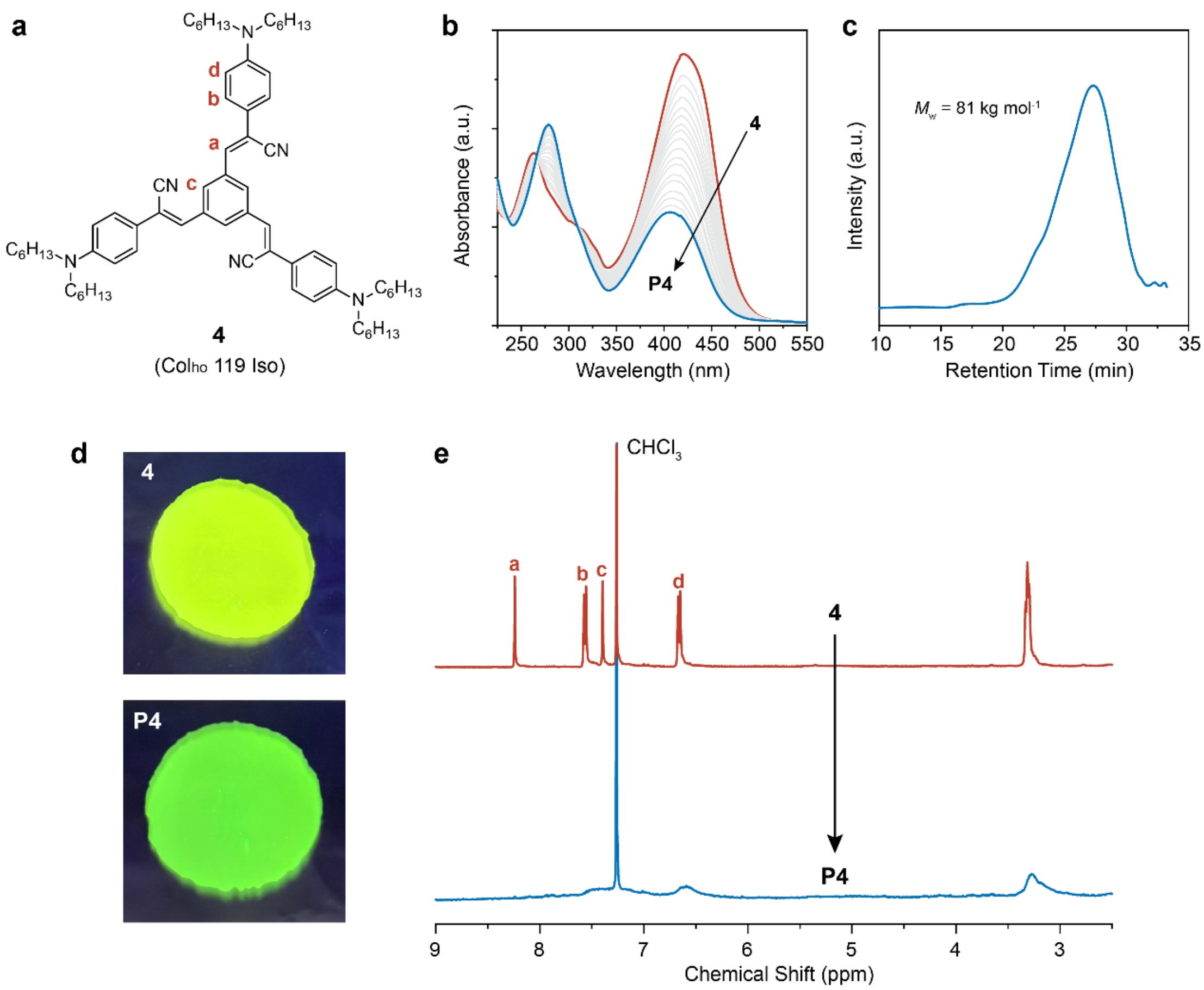

Figure S16. Light-activated TCP of **4** at 50°C in Colho phase. (a) Chemical structure of **4**, bearing three peripheral dialkylamino chains, along with its thermotropic phase behavior (adapted from our previous work [5]). (b) UV-vis absorption spectra of a thin film of **4** recorded under 365 nm light irradiation, demonstrating the progression of polymerization. (c) GPC trace of the resulting polymer **P4**. (d) Fluorescence images of **4** and **P4** following light-activated TCP. (e) ^{1}H NMR spectra of **4** and **P4** in $CDCl_3$.

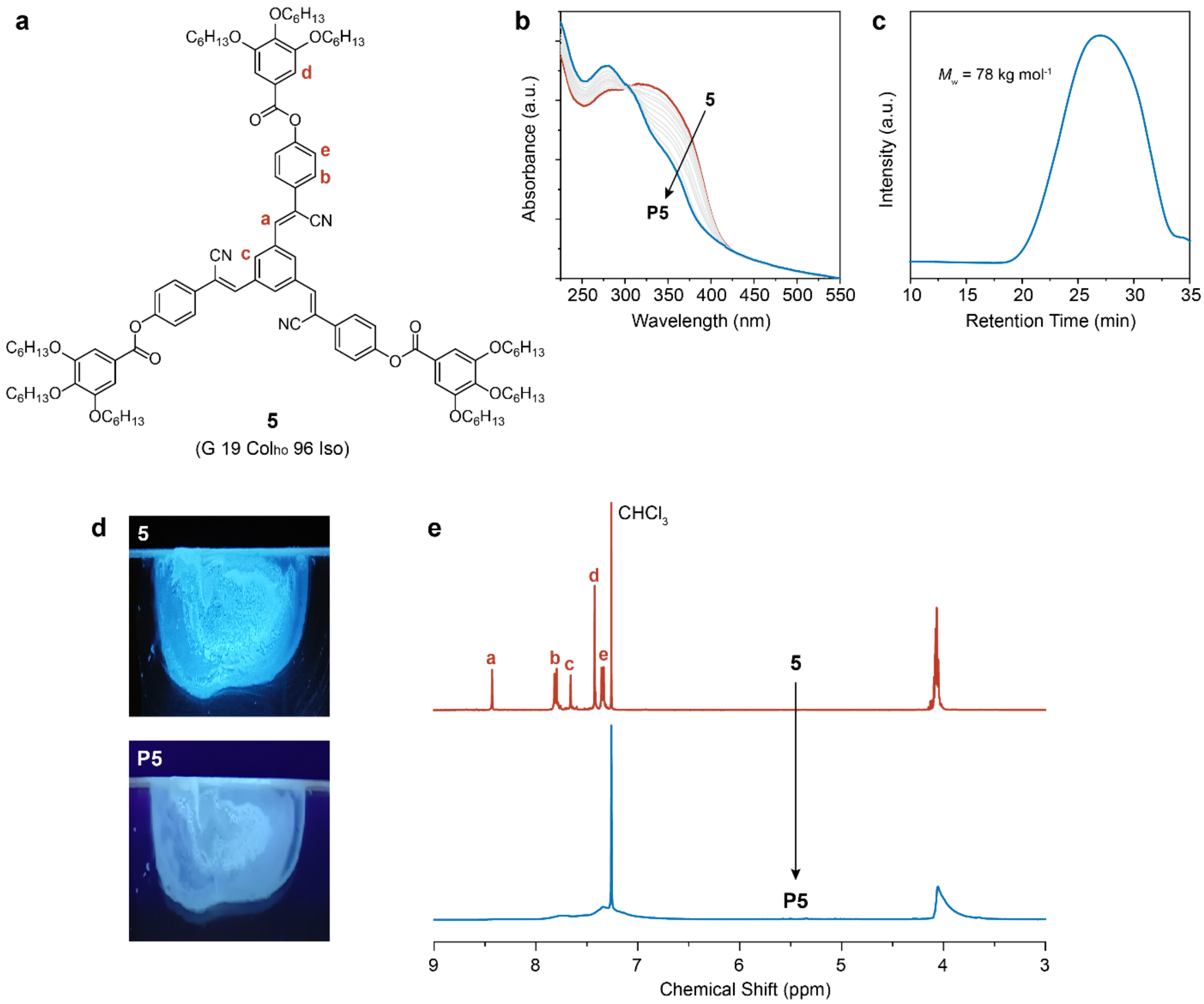

Figure S17. Light-activated TCP of **5** at 45°C in Col_{ho} phase. (a) Chemical structure of **5**, bearing three wedge-shaped trialkyloxyphenyl groups linked via ester bond, along with its thermotropic phase behavior (adapted from our previous work [6]). (b) UV-vis absorption spectra of a thin film of **5** recorded under 365 nm light irradiation, demonstrating the progression of polymerization. (c) GPC trace of the resulting polymer **P5**. (d) Fluorescence images of **5** and **P5** following light-activated TCP. (e) ^{1}H NMR spectra of **5** and **P5** in $CDCl_3$.

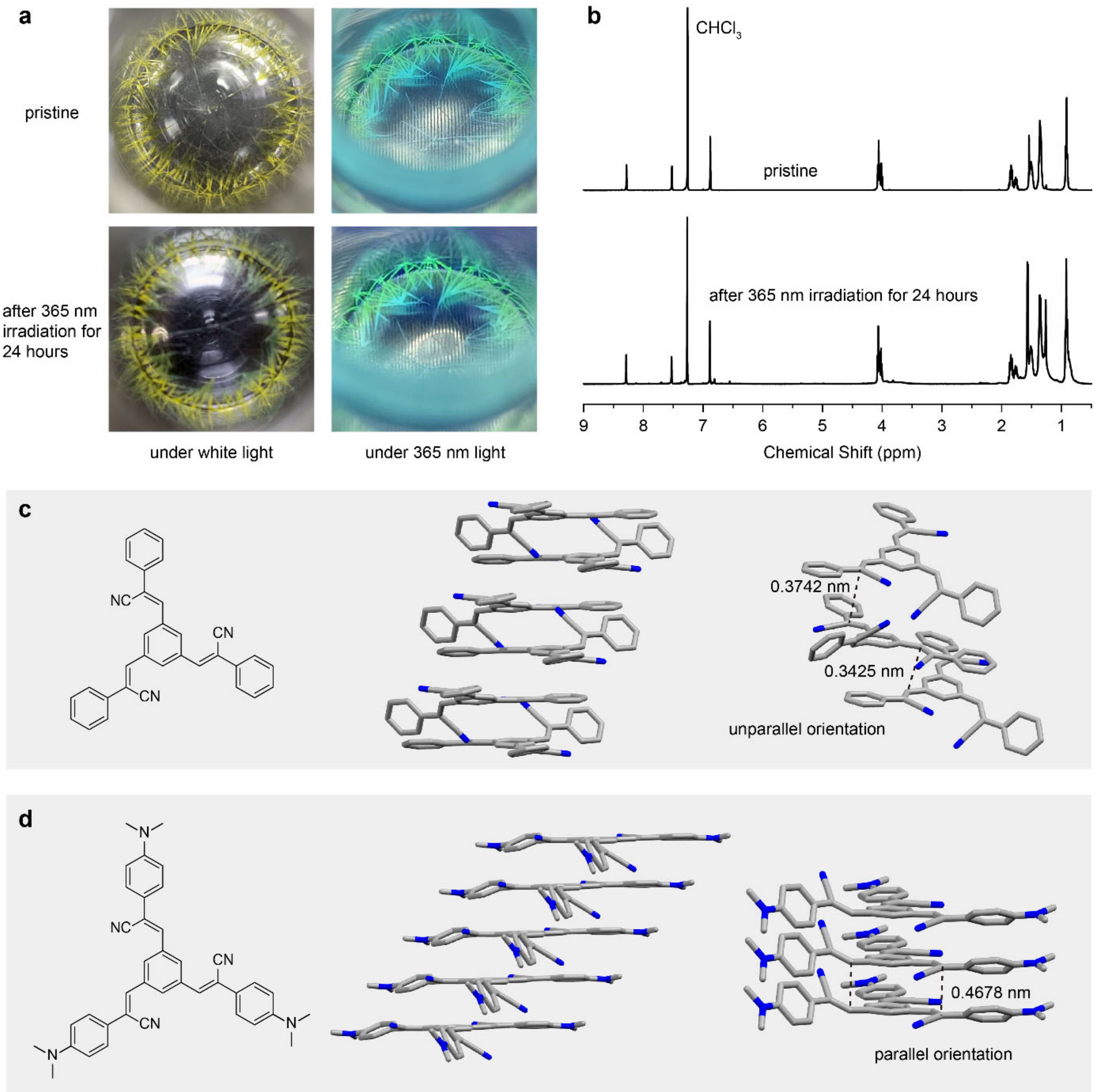

Figure S18. Photochemical stability of **1** in Cr phase. (a) Photographs of crystal **1** before and after 365 nm light irradiation for 24 hours. The needle-like crystals were prepared by slow evaporation of a dichloromethane solution of **1**. (b) Comparison of the ^{1}H NMR spectra of crystal **1** before and after 365 nm light irradiation for 24 hours, demonstrating that **1** remains photochemically stable in its low-temperature Cr phase. (c) Single-crystal structure of the control CS compound without alkoxy substitution (CCDC 1962647, adapted from our previous work [6]). (d) Single-crystal structure of the control CS compound with dimethylamino substitution (CCDC 2224290, adapted from our previous work [5]).

Photochemical Stability in Crystalline Phase. We attempted to grow single crystal of monomer **1**; however, these efforts were unsuccessful. The obtained needle-like crystals were too tenuous and fragile for single crystal X-ray diffraction analysis (Fig. S18a), likely due to the presence of bulky alkyl substituents. Nevertheless, SAXS and WAXS profiles allowed us to propose a rectangular columnar crystalline (Cr) phase (Fig. S7). In this Cr phase, pronounced geometrical constraints hindered the occurrence of TCP. As a result, no significant changes were observed in photographs taken under white light and 365 nm UV light, nor in the ^{1}H NMR spectra after 24 hours of UV irradiation (Fig. S18a,b). These geometry constraints are further supported by the single crystal structures of related control compounds bearing the same three-armed CS core (Fig. S18c,d). Although both control compounds adopt columnar molecular packing, their geometrical parameters do not satisfy the requirements for TCP. These observations highlight the crucial role of dynamic LC order in activating TCP and overcoming the geometric constraints inherent in solid-state synthesis.

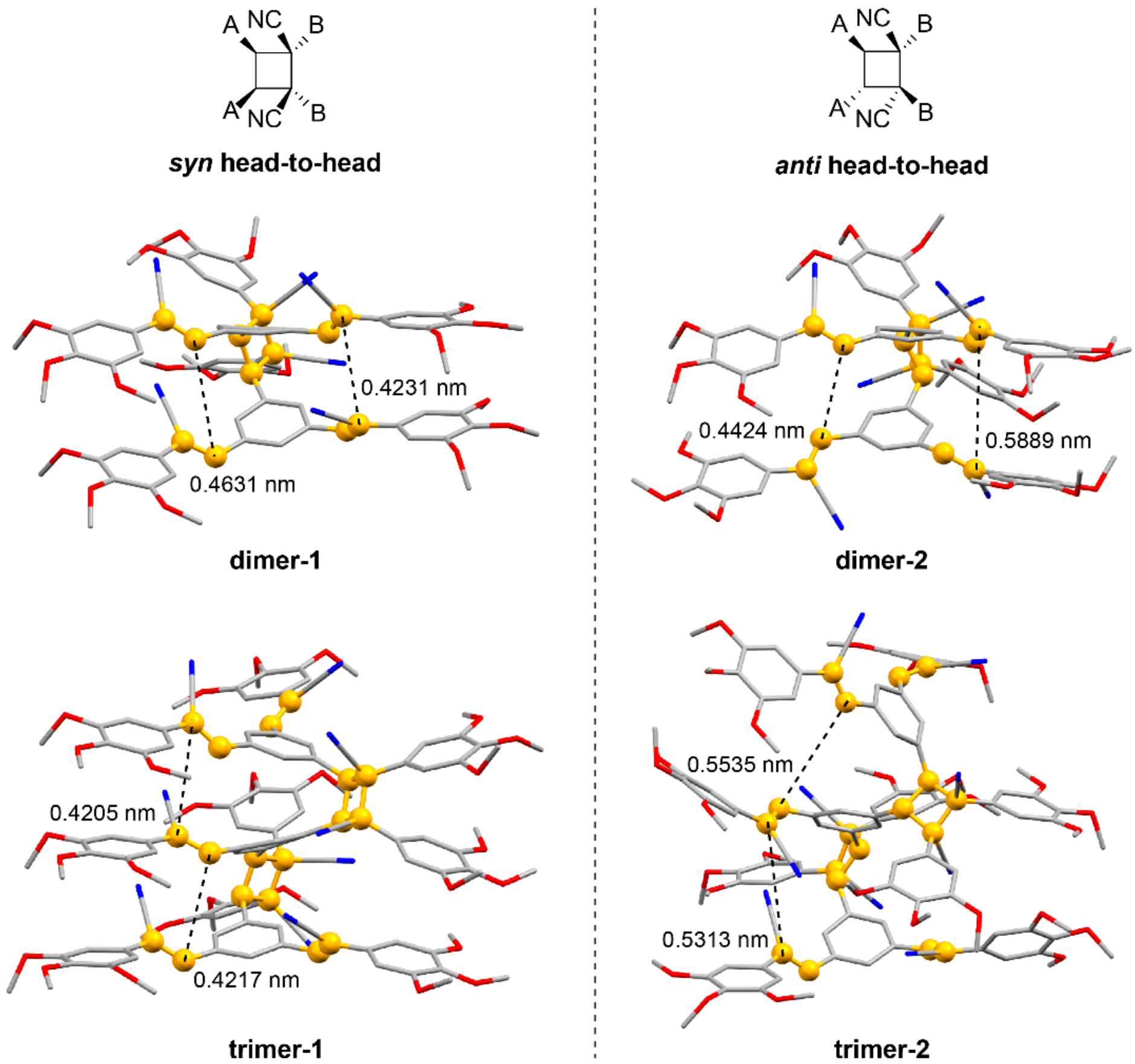

Figure S19. Optimized geometries of the dimer and trimer of **1** after TCP. The anisotropic columnar organization constraints intermolecular packing, leading to position-specific [2 + 2] cycloaddition and the formation of head-to-head cyclobutane structures. Due to stereoisomerism, two distinct isomers may form. To account for this, both plausible [2 + 2] cycloaddition pathways for the dimer and trimer were energy-optimized using DFT with the B3LYP hybrid functional and the 6-311G(d) basis set. For clarity, the nine hexyloxy chains on the peripheral benzene rings were replaced with methoxy groups.

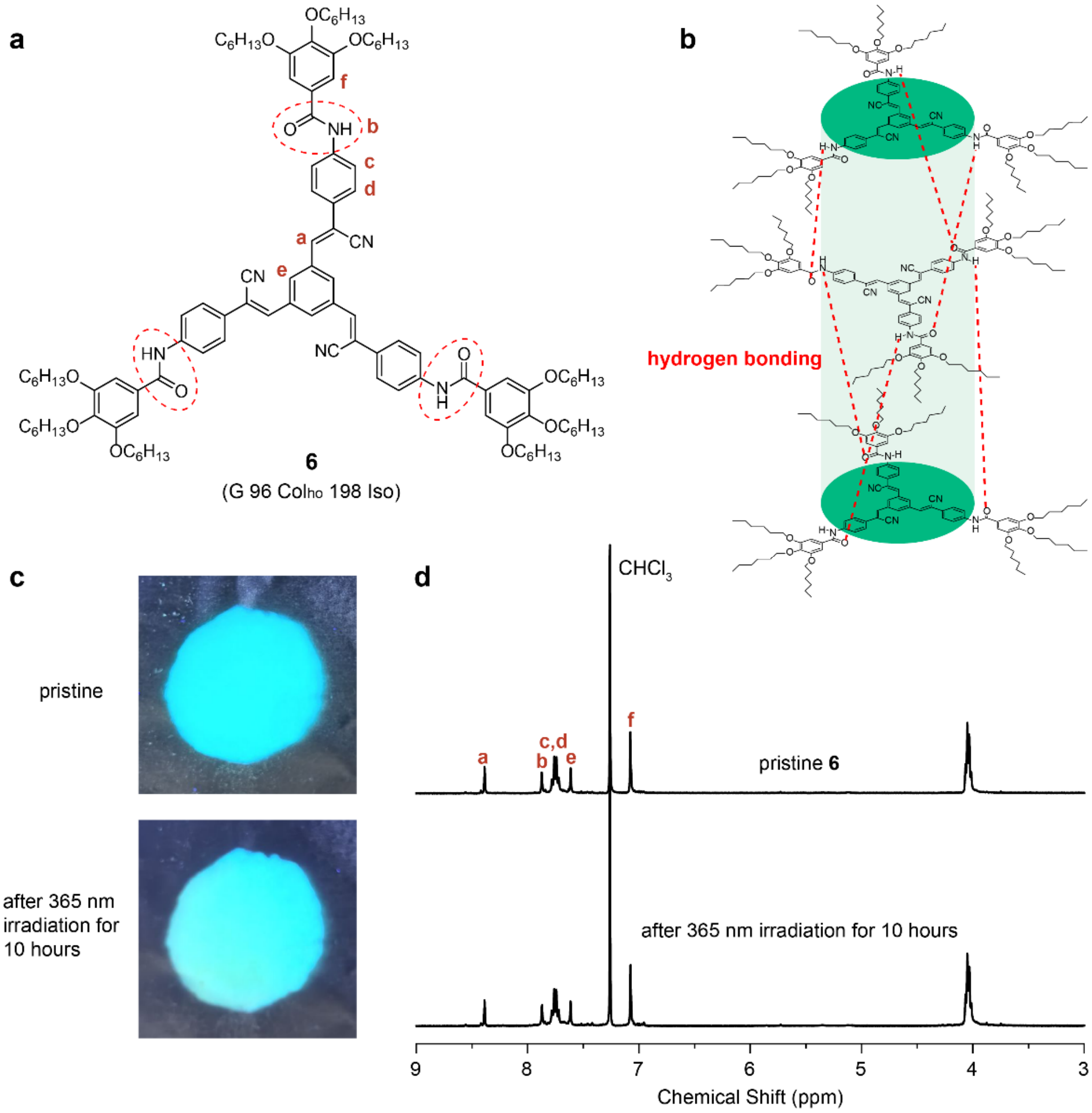

Figure S20. Photochemical stability of **6** in Colho phase. (a) Chemical structure of **6**, bearing three wedge-shaped trialkyloxyphenyl groups linked via amide bond, along with its thermotropic behavior (adapted from our previous work [6]). (b) Schematic illustration of the hydrogen-bonding manner within a LC column. (c) Fluorescence images of **6** before and after 10 hours of 365 nm light irradiation at 120°C. (d) Comparison of the ^{1}H NMR spectra of **6** before and after 10 hours of 365 nm light irradiation at 120°C. These results confirm that **6** remains photochemically stable in the LC state, due to the restricted molecular mobility imposed by intermolecular hydrogen bonding.

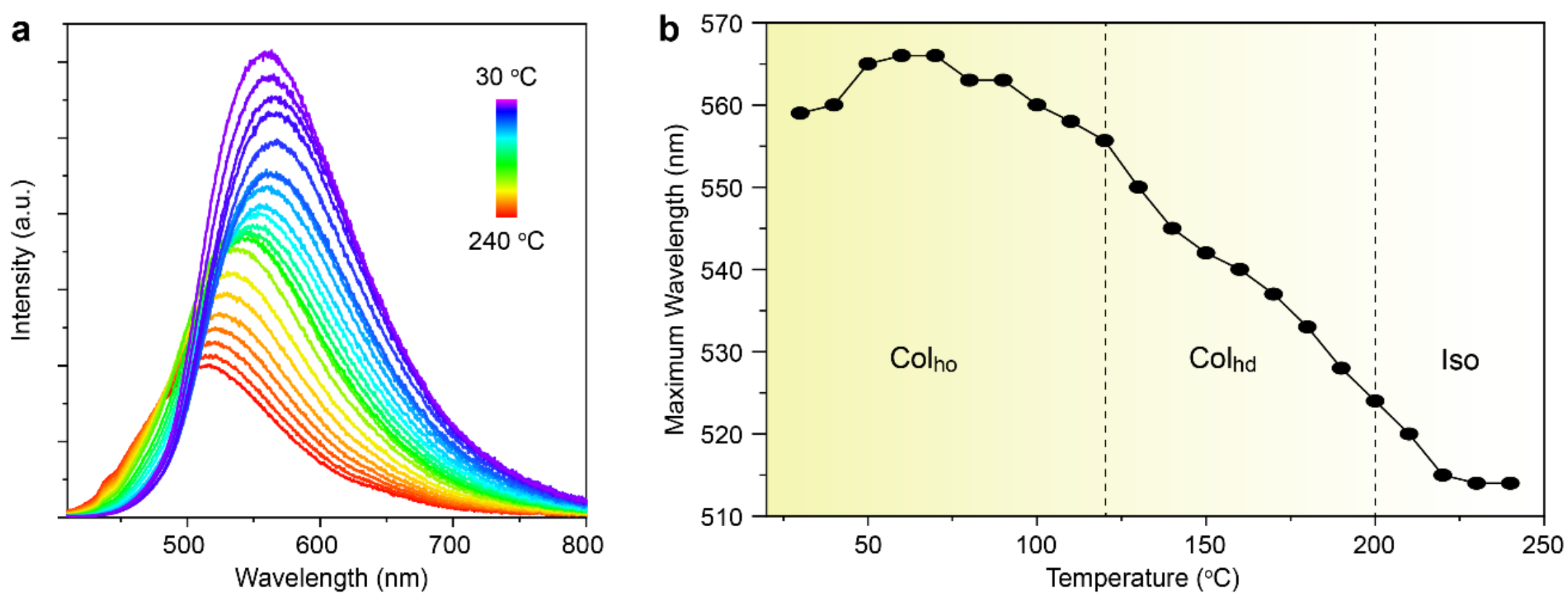

Figure S21. Temperature-dependent fluorescence properties of **1**. (a) Fluorescence emission spectra recorded from 30 to 240°C with an interval of 10°C. (b) Plot of the maximum emission wavelength as a function of temperature. Vertical dashed lines represent the phase transition temperature determined by DSC analysis.

Molecular Dynamics Differences Between Col_{ho} and Col_{hd}. The photophysical properties of molecular systems in the condensed phase, such as fluorescence emission, are highly sensitive to molecular stacking, as intermolecular interactions significantly influence photophysical processes. In this context, in situ fluorescence spectroscopy was employed to monitor the evolution of molecular packing structures. As shown in Fig. S21a, the fluorescence emission intensity decreased and the emission wavelength blue-shifted with increasing temperature. By correlating the maximum emission wavelength with temperature (Fig. S21b), three distinct regions were identified, corresponding to phase transitions observed by DSC. In the Col_{ho} phase, the emission maximum remained around 560 nm with minimal shift, consistent with a highly ordered helical columnar structure. In contrast, a pronounced blue shift from 560 nm to 520 nm was observed in the Col_{hd} phase upon heating. This divergence suggests significantly different molecular packing arrangements between the two phases. The sharp spectral change in the Col_{hd} phase likely arises from increased molecular disorder and mobility within the columns, leading to stronger temperature dependence of the emission properties.

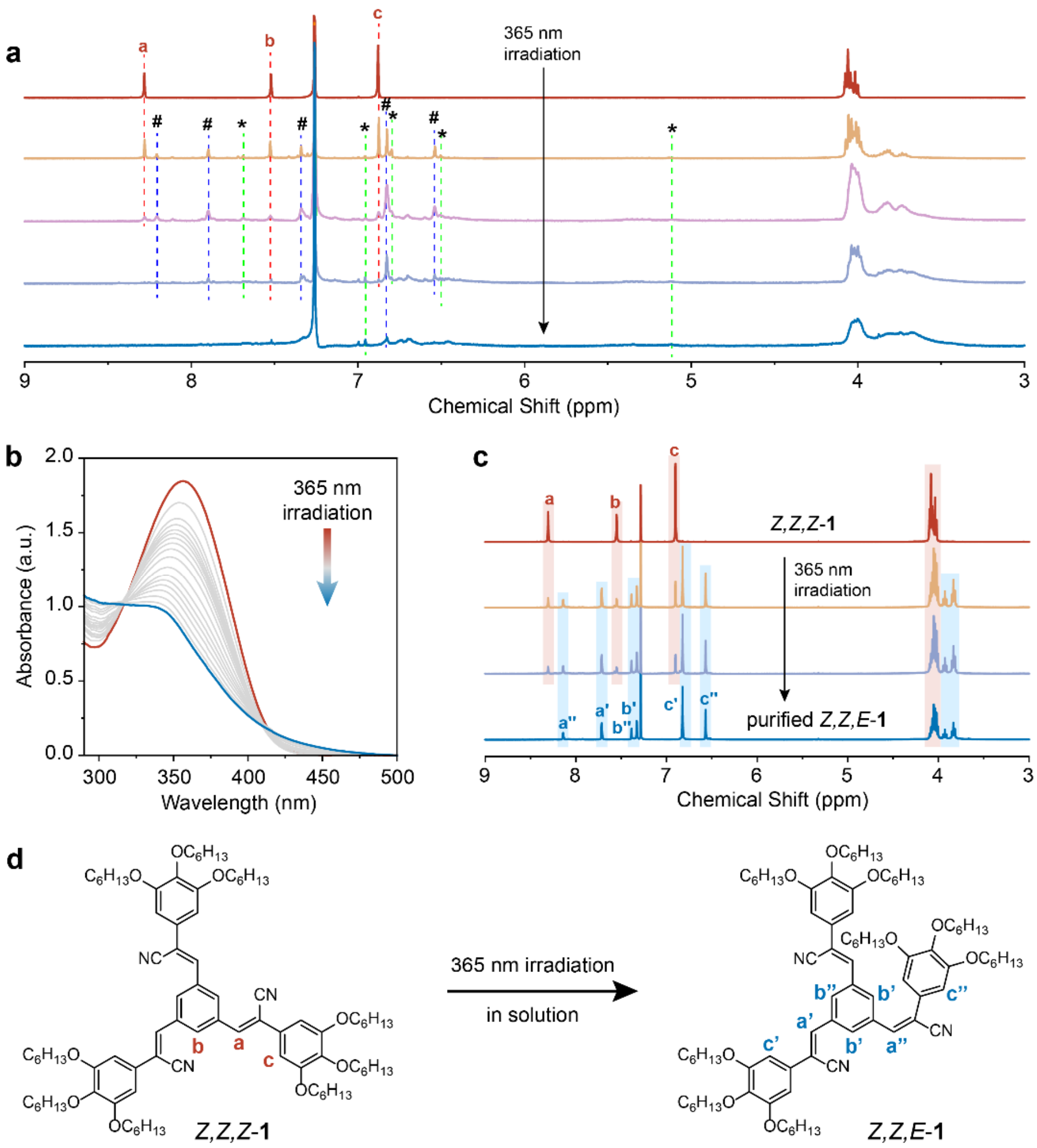

Figure S22. Chemical structure change of **1** during TCP at 175°C in Col_{hd} phase. (a) The evolution of ^{1}H NMR spectra in $CDCl_3$, showing the chemical structure change of **1** upon 365 nm light irradiation at 175°C in the Col_{hd} phase. The hash symbol (#) denotes photoisomerization events, while the asterisk (*) indicates TCP via [2 + 2] cycloaddition. (b) The evolution of UV-vis absorption spectra, and (c) the evolution of ^{1}H NMR spectra in solution, highlighting light-induced Z-E isomerization of one of the three CS arms of **1**, as illustrated in (d).

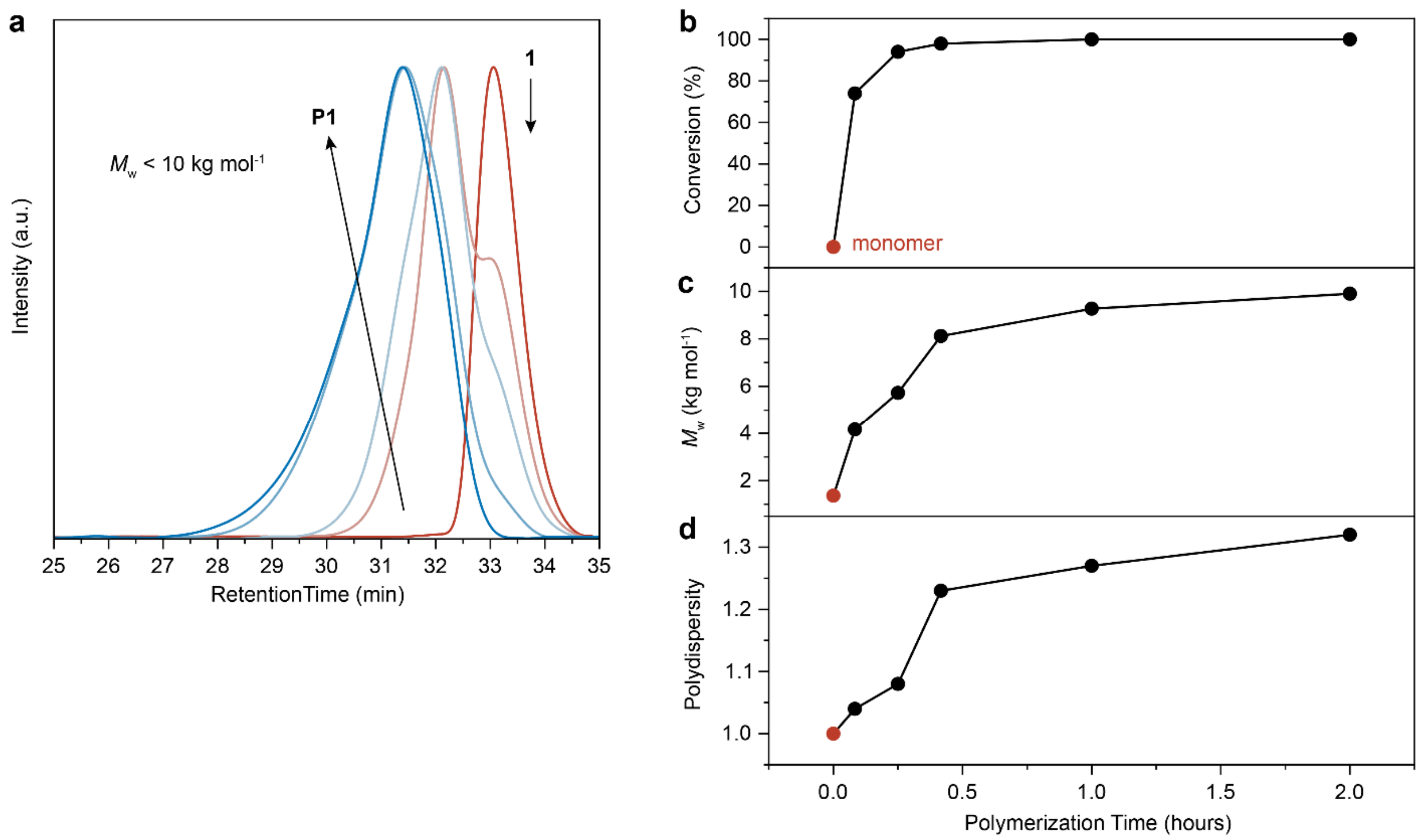

Figure S23. Light-activated TCP parameters of **1** at 175°C. (a) The evolution of GPC traces, showing increased monomer conversion and increased molecular weight with prolonged 365 nm irradiation. (b) Monomer conversion, (c) weight-average molecular weight (M_w), and (d) polydispersity index obtained from GPC analysis of polymers synthesized via TCP at 175°C with varying polymerization time. The red point indicates monomer **1**.

TCP in Col$_{hd}$ Phase. To explore the role of dynamic LC ordering in TCP, we performed light-activated polymerization of **1** at 175°C in the Col$_{hd}$ phase. The reaction was monitored by ^{1}H NMR spectroscopy, which revealed a greater number of resonance peaks compared to the polymerization carried out at 75°C (Fig. S22a). These additional peaks are attributed to Z-E isomerization, likely facilitated by the increased molecular mobility in the Col$_{hd}$ phase. To confirm this, we performed a photochemistry experiment of monomer **1** in solution, where only Z-E isomerization of the CS cores occur. As expected, Z-E isomerization of one of the three CS arms was observed (Fig. S22b-d). Comparative spectral analysis indicated that both [2 + 2] cycloaddition and Z-E isomerization took place concurrently during TCP in the Col$_{hd}$ phase. The formation of the twisted E-isomer hindered further chain growth, resulting in the formation of only low molecular weight oligomers (Fig. S23).

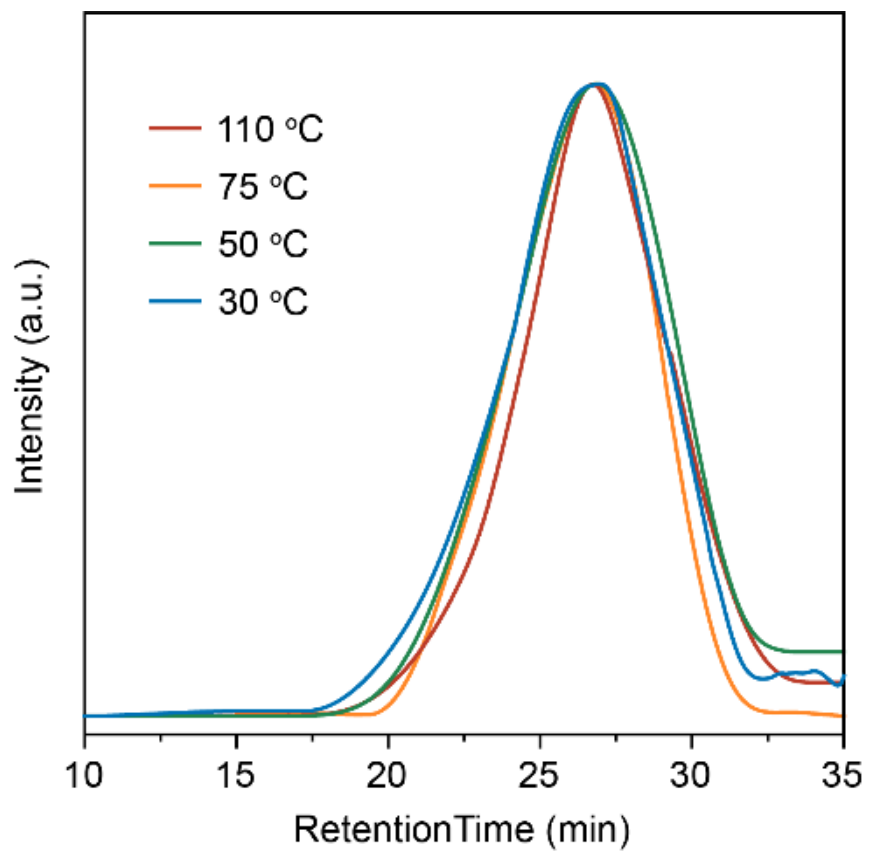

Figure S24. Light-activated TCP of **1** in Col_{ho} phase. GPC traces of light-activated TCP at various temperatures in the Col_{ho} phase, showing little temperature dependence of the molecular weights and polydispersity.

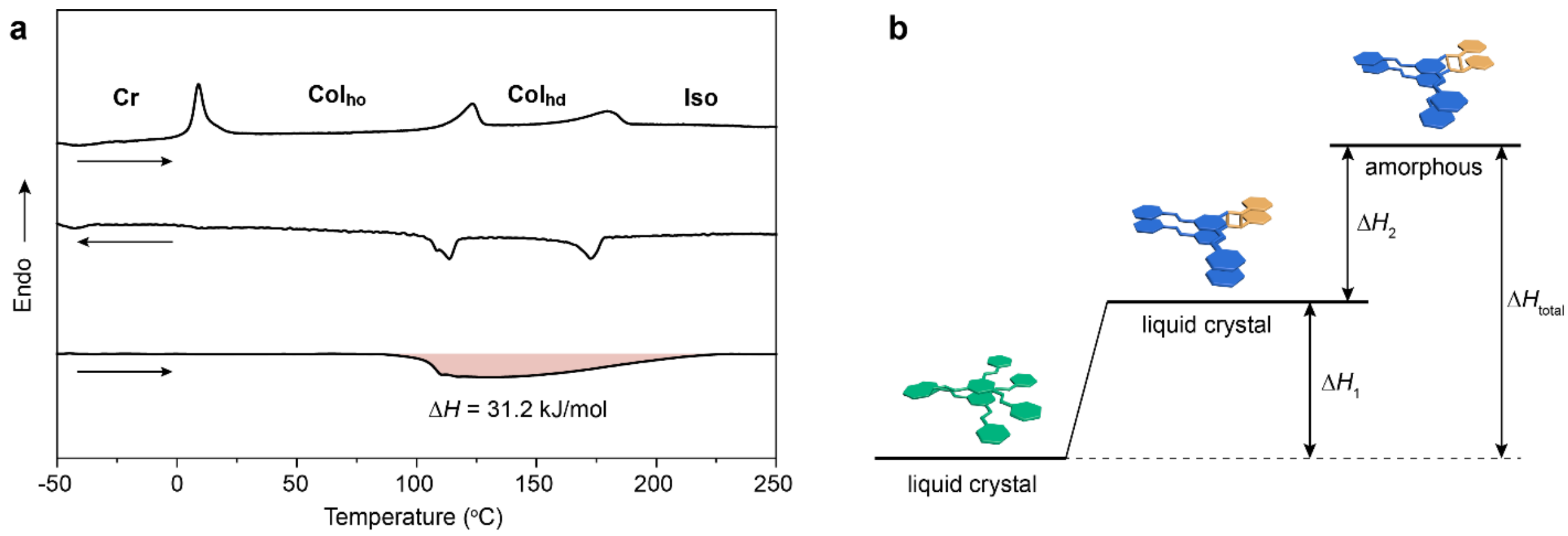

Figure S25. Depolymerization process of **P1**. (a) DSC of **P1** during the first two heating and cooling cycles. (b) Illustration of the energy levels during thermal reversion. ΔH_1, cycloaddition energy; ΔH_2, liquid crystallization energy; ΔH_{total}, total energy. The depolymerization is achieved by thermal relaxation, releasing the energy difference between the thermodynamically stable monomer and metastable polymer in the form of heat. DSC analysis revealed a broad exothermic transition upon heating above 100°C, corresponding to an energy release of approximately 31.2 kJ mol^{-1}. The subsequent cooling and second heating cycle exhibit Cr-Col$_{ho}$-Col$_{hd}$-Iso transitions, which closely match those of the monomer **1**.

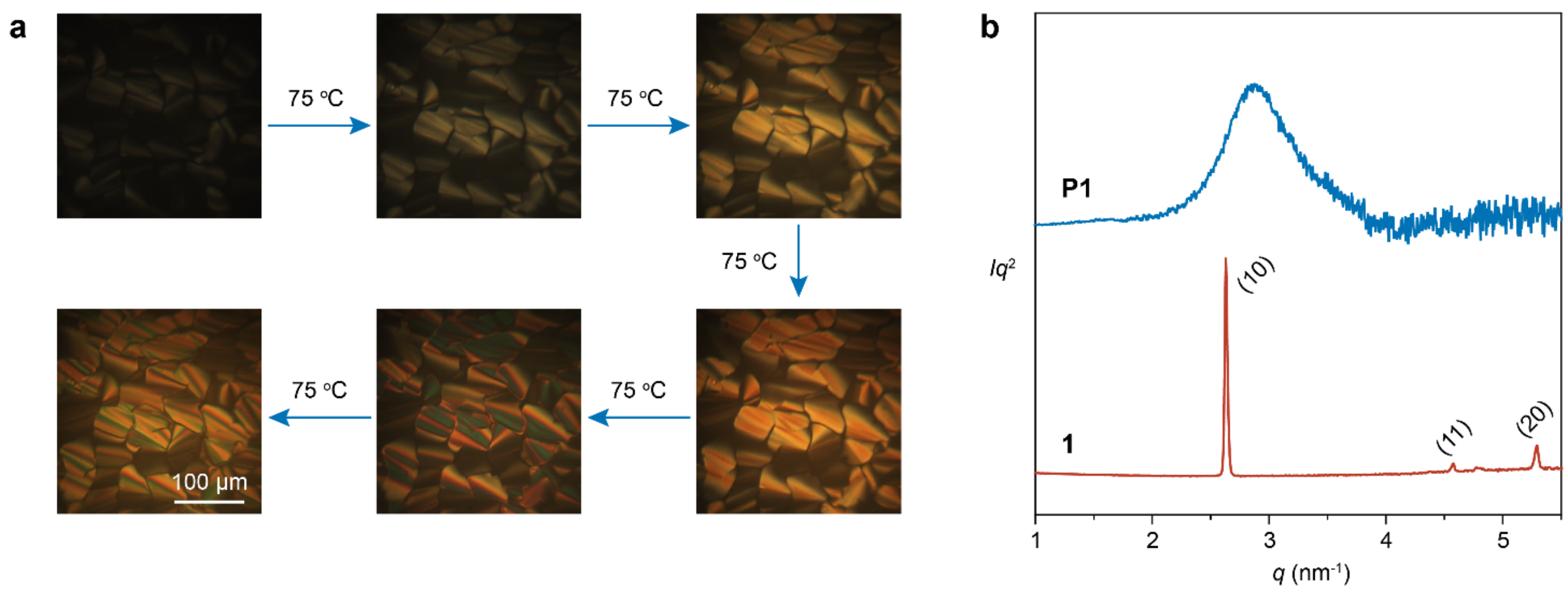

Figure S26. Structural reversion during depolymerization of **P1** at 75°C. (a) POM images showing the gradual increase in columnar LC order during thermal depolymerization of **P1** at 75°C. (b) SAXS profiles of **P1** and the regenerated monomer **1** after complete depolymerization, illustrating the transition from a weakly ordered columnar phase to a highly ordered Col_{ho} structure, along with an increase in columnar dimensions.

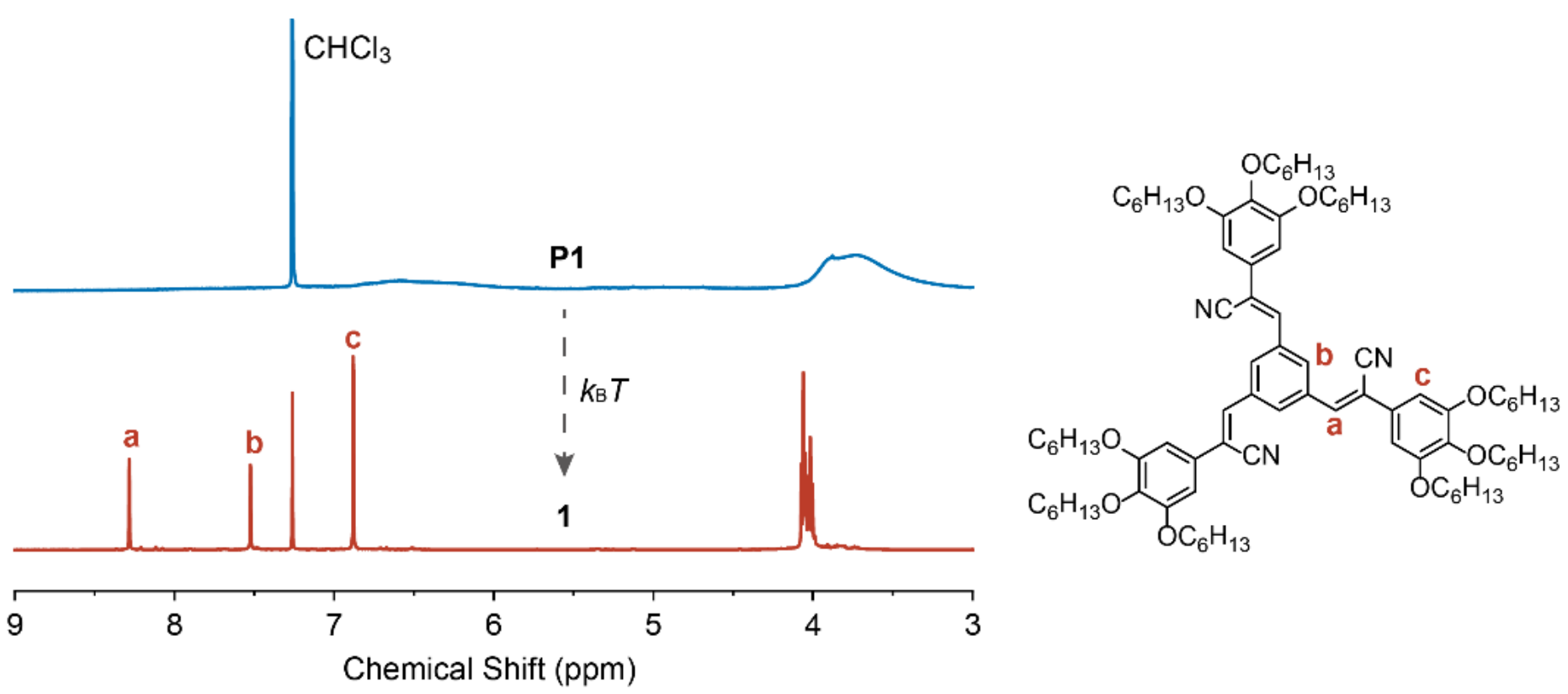

Figure S27. Molecular structure identification of the depolymerization of **P1**. ^{1}H NMR spectra in $CDCl_3$ showing the nearly complete depolymerization of polymer **P1** into monomer **1** after thermal treatment.

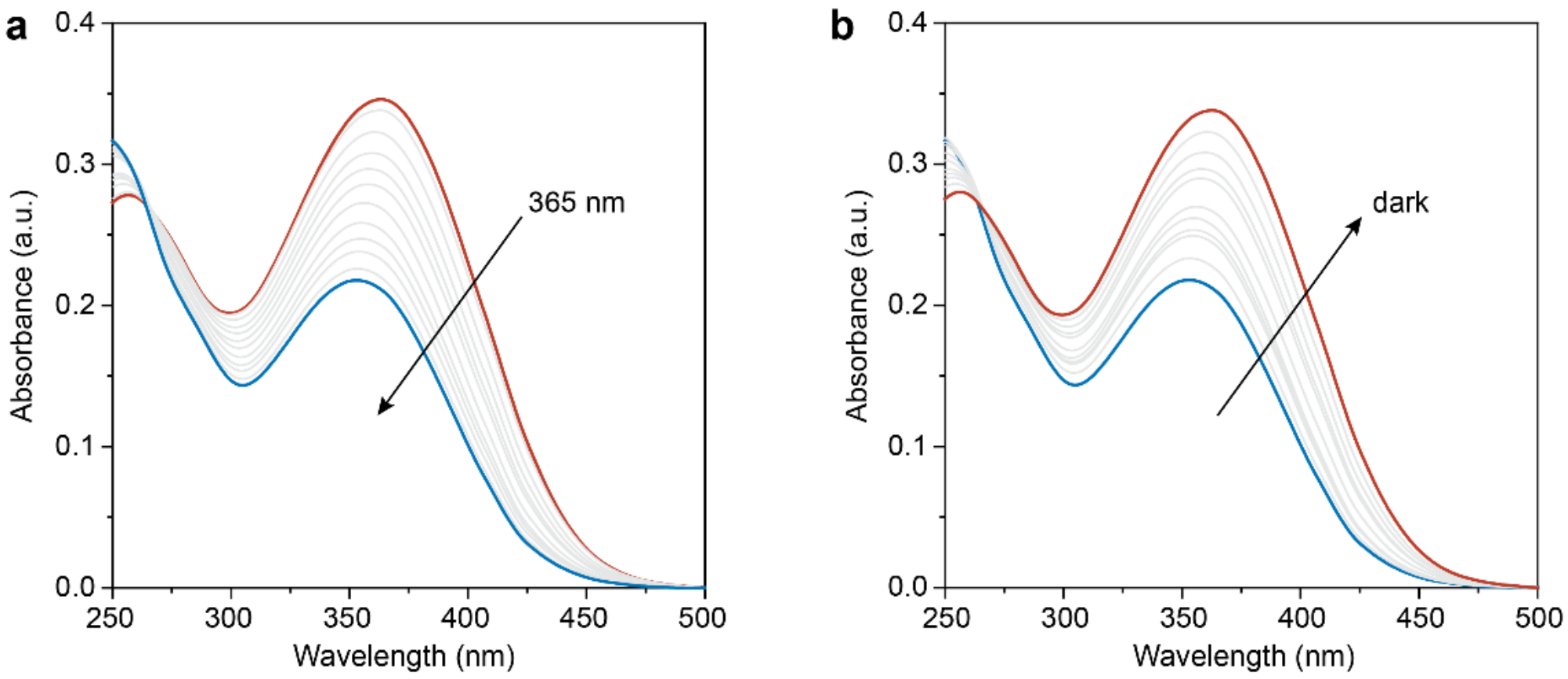

Figure S28. UV-vis spectra illustrating the dissipative TCP process at 75°C. (a) UV-vis absorption spectra recorded during the polymerization of **1** in thin film upon 365 nm light irradiation. (b) UV-vis absorption spectra recorded during the depolymerization of **P1** upon keeping at 75°C.

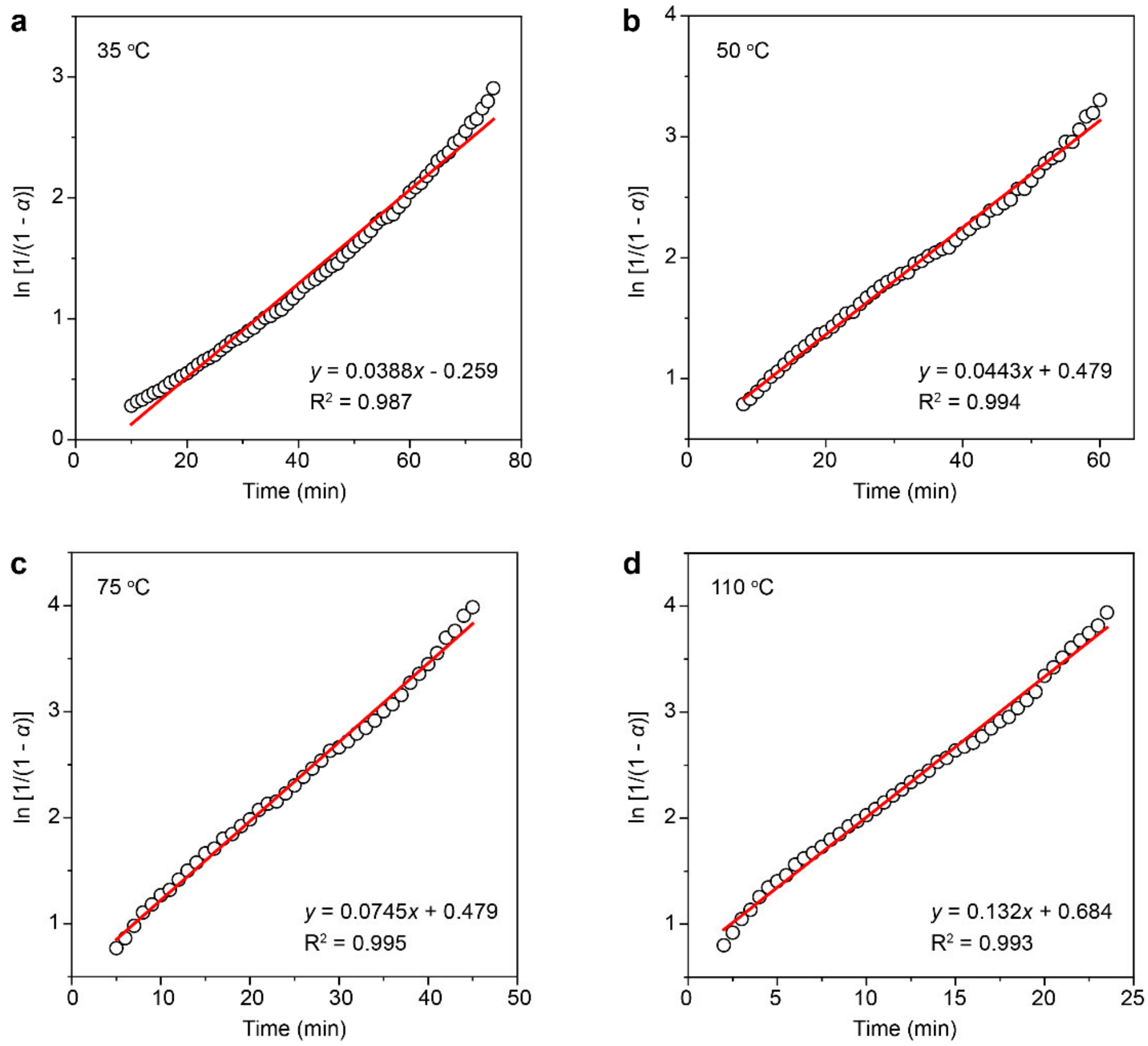

Figure S29. Light-activated TCP kinetics of **1** in Col_{ho} phase. Plots of ln [1/(1–α)] versus time during polymerization at (a) 35°C, (b) 50°C, (**c**) 75°C, and (d) 110°C, where α represents the conversion. The red lines correspond to the linear fit. The data reveal that the TCP follows nearly first-order reaction kinetics, with rate constants of 0.0388 min^{-1}, 0.0443 min^{-1}, 0.0745 min^{-1}, and 0.132 min^{-1} at 35°C, 50°C, 75°C, and 110°C, respectively.

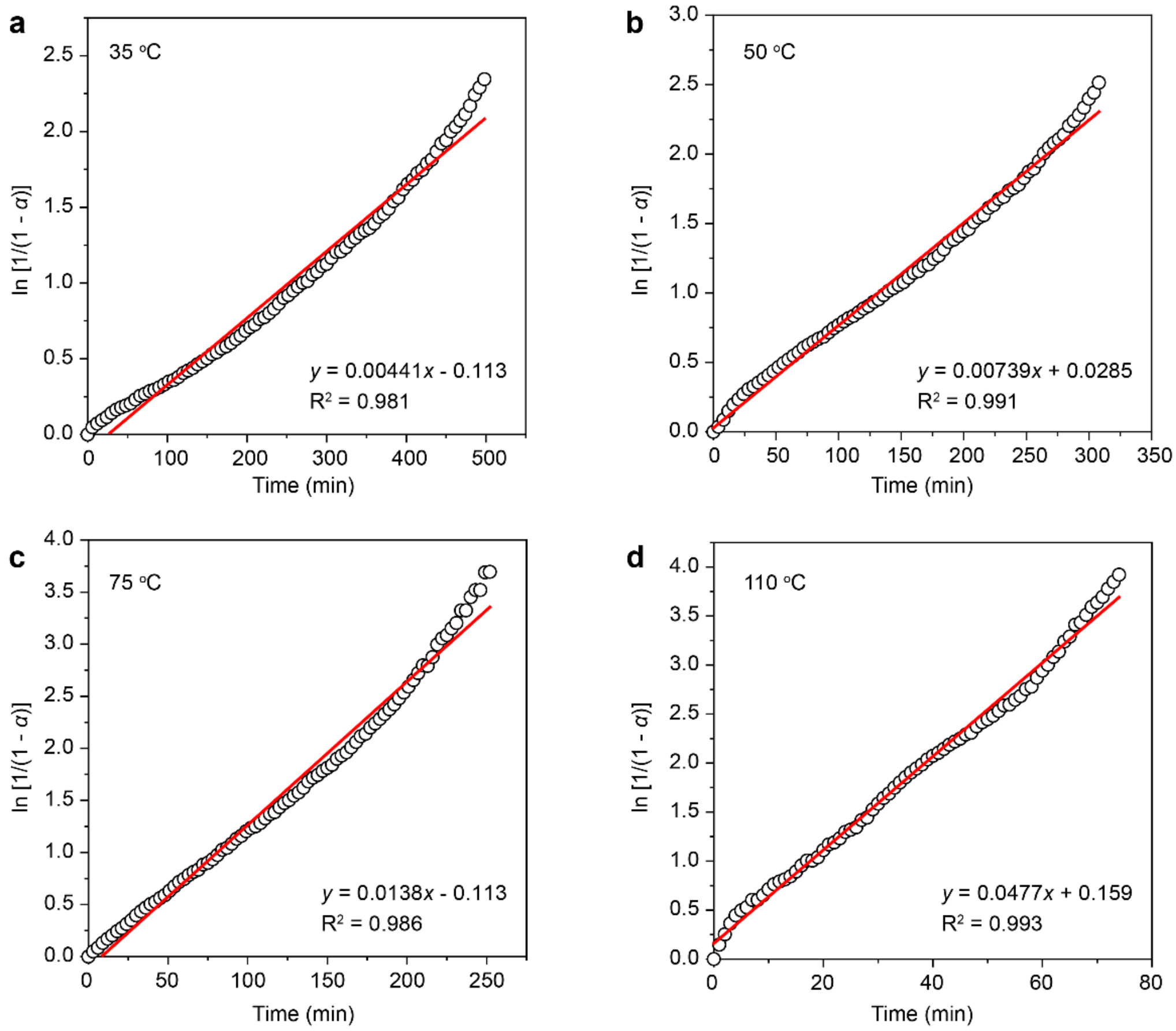

Figure S30. Thermal depolymerization kinetics of **P1**. Plots of ln[1/(1–α)] versus time during depolymerization at (a) 35°C, (b) 50°C, (c) 75°C, and (d) 110°C, where α represents the conversion. The red lines correspond to the linear fit. The data reveal that the depolymerization follows nearly first-order reaction kinetics, with rate constants of 0.00441 min^{-1}, 0.00739 min^{-1}, 0.0138 min^{-1}, and 0.0477 min^{-1} at 35°C, 50°C, 75°C, and 110°C, respectively.

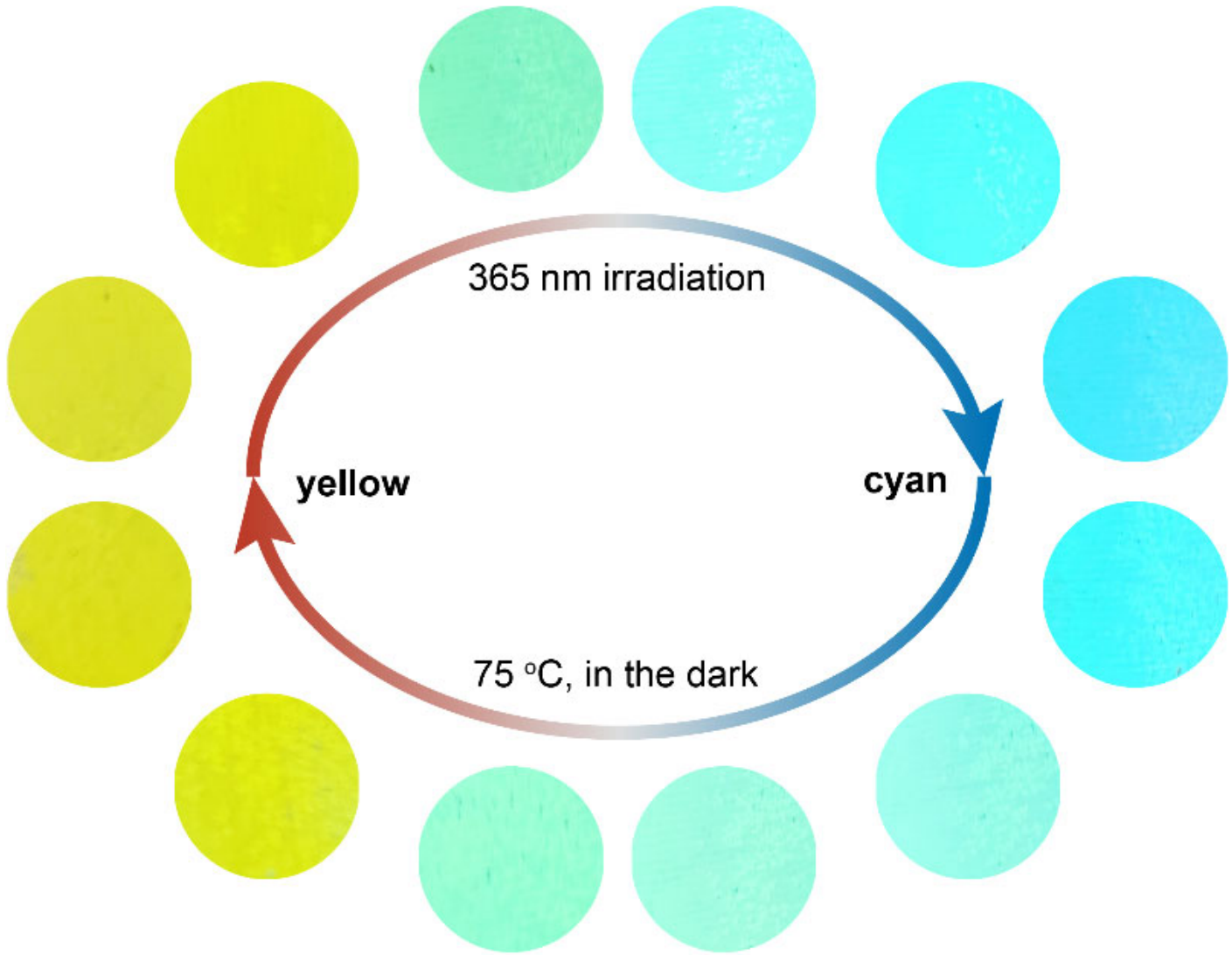

Figure S31. Fluorescence images during dissipative TCP of **1**. The evolution of fluorescent patterns under 365 nm light irradiation at 75°C, followed by time-dependent reversion via temperature-induced self-erasing at 75°C.

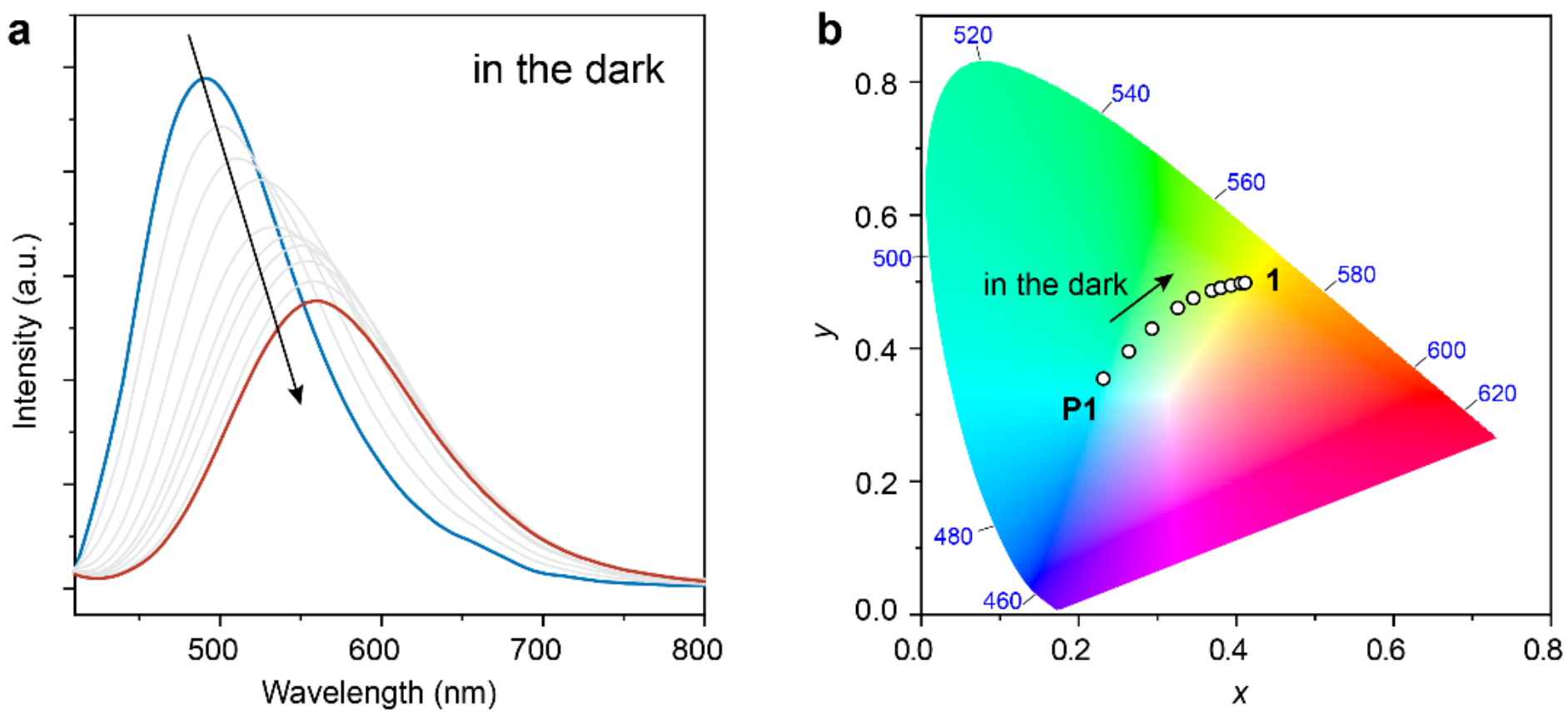

Figure S32. Fluorescence characterization of the depolymerization of **P1** at 75°C. (a) Evolution of fluorescence emission spectra during the depolymerization of **P1** at 75°C in the dark. (b) The corresponding CIE 1931 chromaticity diagram illustrating the color switching trace during depolymerization.

Preparation of LC Films. Quartz slides were thoroughly cleaned by sequential rinsing with ethanol and acetone, followed by drying using an air gun. A 5 wt% aqueous solution of polyacrylic acid was then spin-coated onto the slides at 3,000 rpm for 60 s to form a sacrificial layer. After baking at 100 °C for 5 min, polyimide tape (500 μm thick) was applied to the sides of the slides as spacers. Monomer **1** was carefully poured onto the slides, heated to its melting point, and mixed with Norland optical adhesive NOA65 until fully dissolved. The resulting mixture was covered with another slide to assemble the cell. After cooling and subsequent photo-induced crosslinking, the sacrificial layer was dissolved with water, yielding free-standing polymer dispersed LC films. Prior to photopatterning, the films were thermally treated to depolymerize any residual polymers formed during the photocrosslinking process.

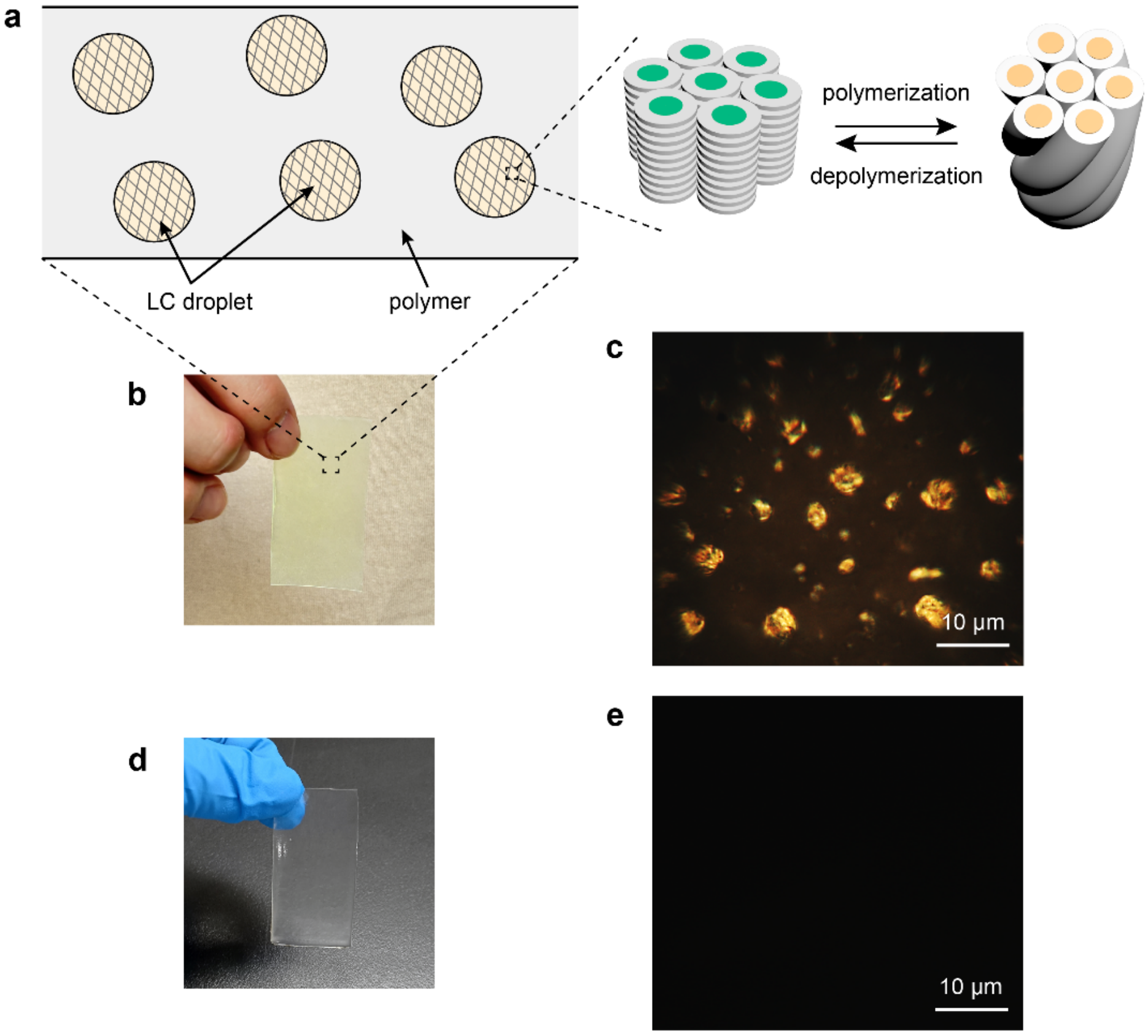

Figure S33. Characterization of the LC films. (a) Cross-sectional view of a polymer dispersed LC thin film, showing LC droplets dispersed within a polymer matrix, where light-induced TCP and thermal depolymerization can occur. (b) Photograph of the as-prepared LC film containing LC monomer **1**. (c) Corresponding POM image demonstrating the ordered LC organization within the droplets. (d) Photograph of the as-prepared film without any dispersed LC materials. (e) Corresponding POM image indicating no birefringence.

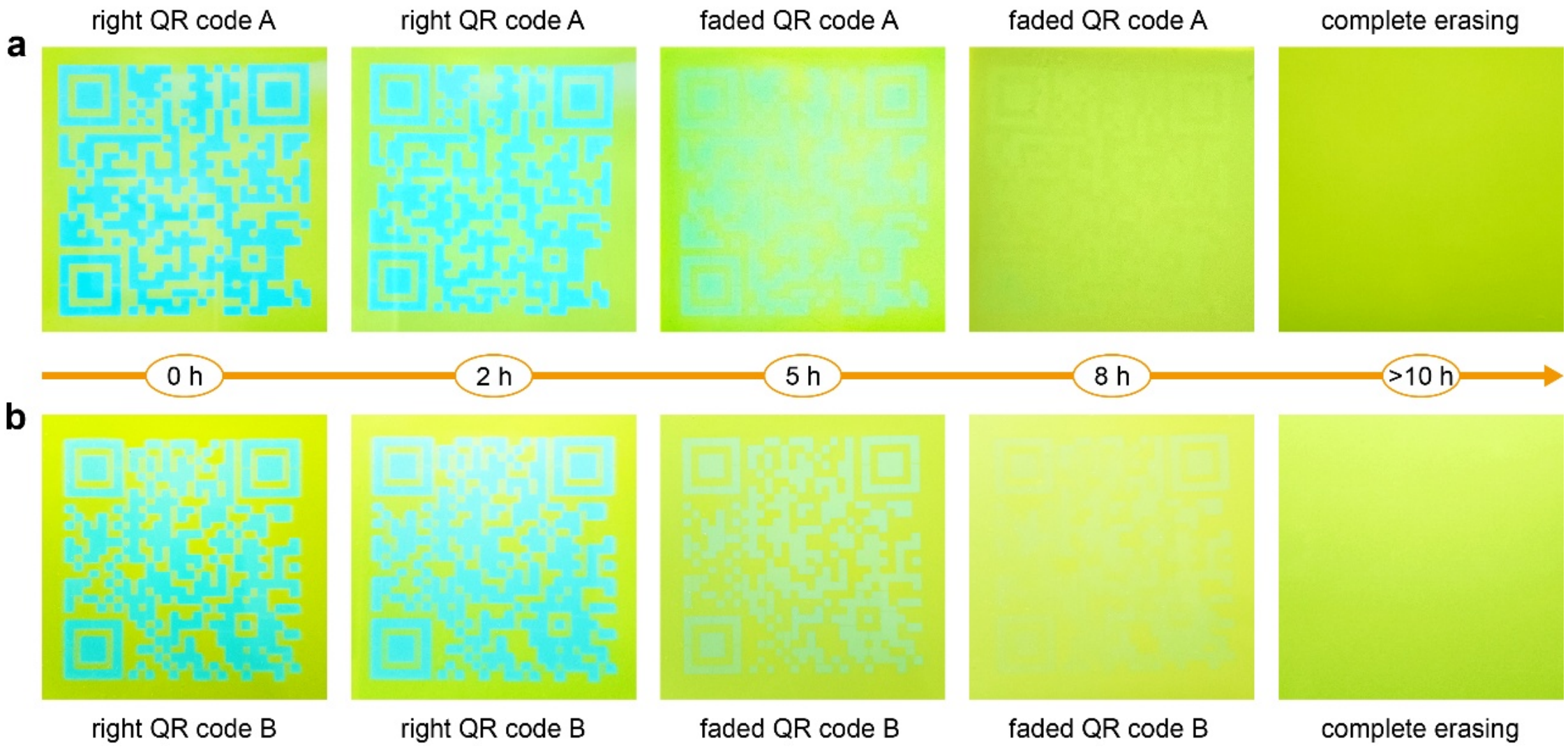

Figure S34. Evolution of fluorescence images of QR code-encoded films. (a) Time-dependent fluorescence changes of a film containing a QR code encoding basic information for student A, upon attachment to human skin. (b) Time-dependent fluorescence changes of a film containing a QR code encoding basic information for student B, upon attachment to human skin.

References

1. Frisch MJ, Trucks GW, Schlegel HB *et al.* Gaussian 16 Rev. C.01; Wallingford CT, 2016.
2. Grimme S, Ehrlich S, Goerigk L. Effect of the damping function in dispersion corrected density functional theory. *J Comput Chem* 2011; **32**: 1456–65.
3. Bannwarth C, Ehlert S, Grimme S. GFN2-xTB-an accurate and broadly parametrized self-consistent tight-binding quantum chemical method with multipole electrostatics and density-dependent dispersion contributions. *J Chem Theory Comput* 2019; **15**: 1652–71.
4. Mu B, Hao X, Luo X *et al.* Bioinspired polymeric supramolecular columns as efficient yet controllable artificial light-harvesting platform. *Nat Commun* 2024; **15**: 903.
5. Mu B, Ma T, Zhang Z *et al.* Thermo-induced bathochromic emission in columnar discotic liquid crystals realized by intramolecular planarization. *Chem Eur J* 2023; **29**: e202300320.
6. Mu B, Zhao Y, Li X *et al.* Enhanced conductivity and thermochromic luminescence in hydrogen bond-stabilized columnar liquid crystals. *ACS Appl Mater Interfaces* 2020; **12**: 9637–45.
7. Pisula W, Feng X, Müllen K. Tuning the columnar organization of discotic polycyclic aromatic hydrocarbons. *Adv Mater* 2010; **22**: 3634–49.
